# Nanotechnology in Drug Delivery: Anatomy and Molecular Insight into the Self-Assembly of Peptide-Based Hydrogels

**DOI:** 10.3390/molecules29235654

**Published:** 2024-11-29

**Authors:** Adelaide R. Mashweu, Vladimir A. Azov

**Affiliations:** Department of Chemistry, University of the Free State, P.O. Box 339, Bloemfontein 9300, South Africa

**Keywords:** drug delivery, nanocarriers, hydrogels, peptides, peptide folding

## Abstract

The bioavailability, release, and stability of pharmaceuticals under physicochemical conditions is the major cause of drug candidates failing during their clinical trials. Therefore, extensive efforts have been invested in the development of novel drug delivery systems that are able to transport drugs to a desired site and improve bioavailability. Hydrogels, and peptide hydrogels in particular, have been extensively investigated due to their excellent biocompatibility and biodegradability properties. However, peptide hydrogels often have weak mechanical strength, which limits their therapeutic efficacy. Therefore, a number of methods for improving their rheological properties have been established. This review will cover the broad area of drug delivery, focusing on the recent developments in this research field. We will discuss the variety of different types of nanocarrier drug delivery systems and then, more specifically, the significance and perspectives of peptide-based hydrogels. In particular, the interplay of intermolecular forces that govern the self-assembly of peptide hydrogels, progress made in understanding the distinct morphologies of hydrogels, and applications of non-canonical amino acids in hydrogel design will be discussed in more detail.

## 1. Introduction

Drug discovery is an area that is heavily investing in the pursuit of discovering new APIs to combat various diseases. Thousands of drugs known and used are evidence of this achievement. However, the therapeutic efficiency of many is still low due to a number of limitations, such as poor solubility, proteolytic degradation, and short half-lives in circulation due to instability. This is reflected by the fact that 90% of drugs that undergo clinical trials never reach the market [1]. The development of a new API is a time-consuming and expensive process that may take 10–17 years to reach the market [2]. Therefore, despite significant advances and investments in drug discovery, there are still barriers that limit the full potential of APIs and restrict their implementation in the market.

The therapeutic efficiency of new or old drugs may be significantly improved by the introduction of novel drug delivery systems. Drug delivery is defined as approaches or processes of administering pharmaceutical compounds to achieve a therapeutic effect [3,4,5]. The enteral and parenteral routes are conventional modes of administering drugs [6]. The enteral route of administration entails oral, rectal, buccal, or sublingual, while the parenteral route of drug administration is through intravenous, intramuscular, transdermal, and subcutaneous [3,6,7,8]. Once a drug is administered, it undergoes absorption, distribution, metabolism, and excretion (ADME) [9]. The enteral route of administration is preferred due to its noninvasive nature; however, the bioavailability of the drug is reduced as the drug undergoes hepatic first-pass metabolism in the liver [6].

Advanced drug delivery systems may include special drug delivery vehicles that improve the efficacy of drugs in various ways, such as protection against degradation, improving permeation to various membranes, and slow sustained release over longer durations, thus limiting the continuous intake of drugs to stimulate a therapeutic effect, which may potentially result in drug resistance. Drug delivery research has been in the spotlight as many delivery vehicles have been developed. One promising way is to use nanocarriers, in particular peptide hydrogels, as delivery vehicles to improve the efficacy of new or already existing therapeutics without altering the structure and desired effects of the API.

Peptide hydrogels represent a class of nanocarriers with high potential because of the diversity of readily available amino acids, which may be used to synthesize various peptide sequences with varying properties. Moreover, such hydrogels are biocompatible as well as biodegradable; therefore, they will not evoke an immune response once administered. However, there are limitations to the applicability of peptide-based hydrogels, such as the stability/strength of the hydrogel, the capacity of drug loading, and the release pattern of the hydrogel.

The distinct morphology of peptide hydrogels is still being investigated to improve their properties and drug-delivery capabilities. Moreover, an in-depth understanding of the self-assembly process will also shed light on other important natural processes, such as the formation and role of amyloids (markers for neurodegenerative diseases), since there is a similarity in the structure of amyloids and peptide hydrogels.

This review will give an overview of different drug delivery systems and then will focus on hydrogels, in particular peptide-based hydrogelators, used in this capacity.

## 2. Conventional Modes of Drug Delivery

### 2.1. Oral Drug Delivery

**Figure 1:**
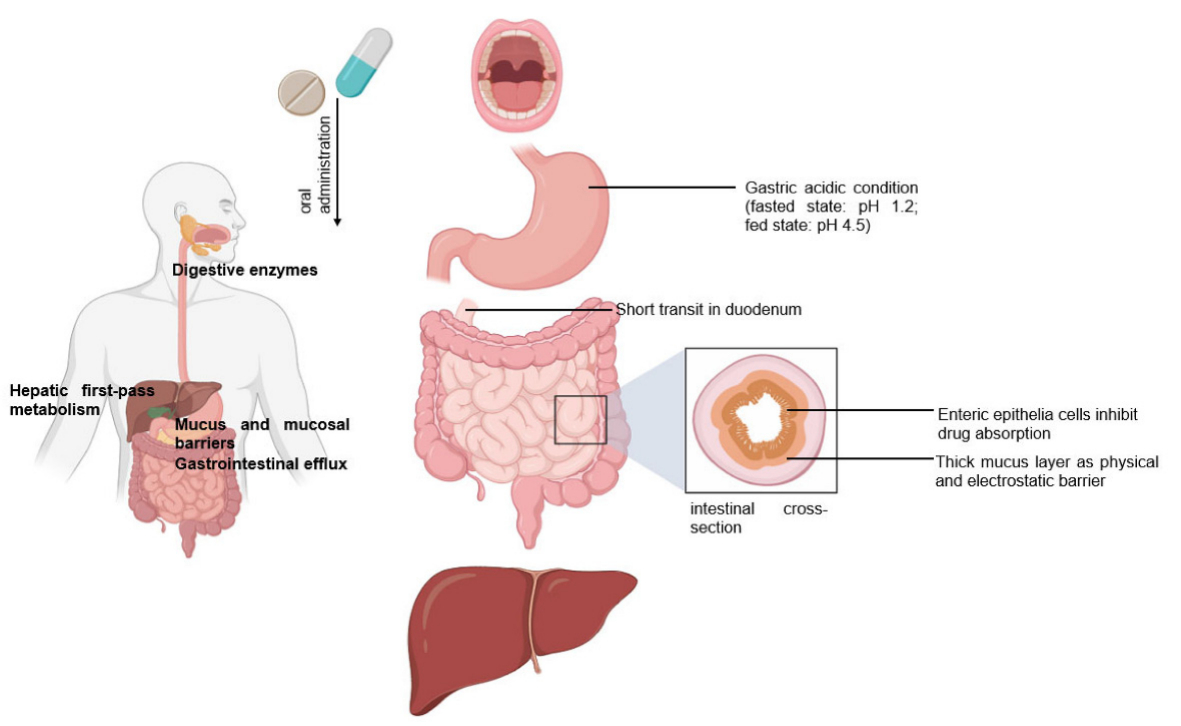
Drug absorption through oral admission. Reprinted with permission from Ref. [10]. Copyright 2023 MDPI.

Oral drug delivery is the most common and convenient mode of administering drugs [10,11,12,13,14]. The drug passes through the gastrointestinal tract, and absorption takes place in the small intestine, where systemic circulation begins [11] (Figure 1). The low pH (pH 1–2) in the gastrointestinal tract and the first-pass metabolism, which takes place in the intestine and liver, resulting in low bioavailability and short circulation times (<12 h) of the administered drug [10,11,14,15]. The majority of therapeutic peptides and proteins are not orally administered due to the rapid degradation in the stomach [16]. Drug absorption via the oral route is slow; as such, it is not preferred during an emergency [17].

### 2.2. Sublingual and Buccal Drug Delivery

Sublingual and buccal routes are considered alternatives to the oral route as they bypass both the first pass metabolism and gastrointestinal tract, and absorption is via the venous drainage to the superior vena cava by placing the drug under the tongue or between the gums and cheeks, respectively, for absorption into the systemic circulation [17,18,19]. This route of drug delivery is especially attractive for highly soluble drugs that undergo hepatic clearance and degradation in the gastrointestinal tract [17,18]. However, not all drugs can be delivered through buccal and sublingual drug delivery [17,18]. The drug may also be prone to swallowing of saliva, which leads to loss of dissolved or suspended drugs [17].

### 2.3. Transdermal Drug Delivery

**Figure 2:**
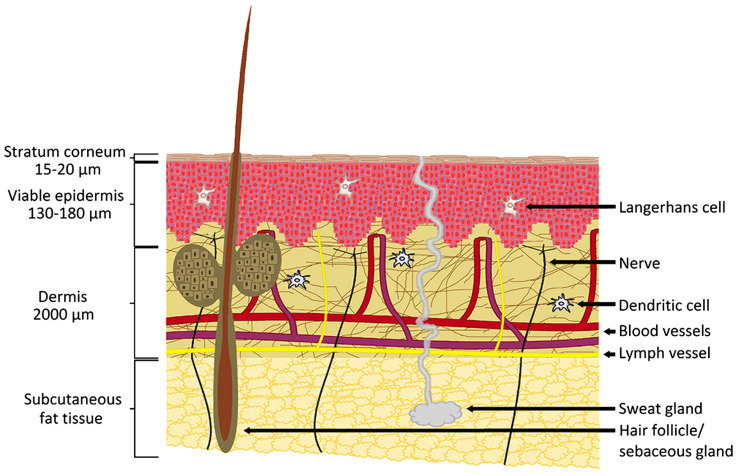
Skin structure depicting different skin layers. Reprinted with permission from Ref. [16]. Copyright 2015 MDPI.

Transdermal drug administration refers to drug absorption through the skin, providing a slow and steady absorption directly into the circulation system [9,20]. The transdermal application of fentanyl or other pain medications (opioids) can provide effective pain management over several hours; a scopolamine patch can relieve motion sickness during the duration of a cruise ship [9]. The drug passes through the epidermis and dermis into the dermal layer, where systemic absorption takes place [16], as shown in Figure 2. The transdermal route of administration is non-invasive, bypassing the gastrointestinal tract and first-pass metabolism [8,16]. Despite the advantages of transdermal administration, there are limitations as the skin absorbs only lipophilic drugs with low MW (less than 1 kDa), and high MW drugs require penetration enhancers for their absorption [8,21].

### 2.4. Intramuscular, Intravenous, and Subcutaneous Drug Delivery

Intramuscular administration is injecting a drug into the depth of selected muscles rich in blood supply, while intravenous (IV) injection is where the drug is injected directly into the veins. Both modes of administration allow for the rapid absorption of the drug, with intravenous administration being the fastest [22,23,24]. Subcutaneous drug delivery is the administration of drugs into the interstitial area underlying the dermis of the skin, where there is slow absorption over an extended period of time as opposed to drugs administered intravenously and intramuscularly [23,24,25,26]. Intramuscular, intravenous, and subcutaneous administration circumvent the gastrointestinal tract and bypass the hepatic first-pass metabolism [22,24]. The primary mode of administering macromolecules, such as proteins, is through intravenous, intramuscular, or subcutaneous administration [16,27]. Subcutaneous injection is the preferred parenteral route of drug administration due to the slow release of drugs, which keeps the plasma drug concentration within the therapeutic window over an extended period of time [21,24,26].

### 2.5. Limitations of Conventional Modes of Drug Delivery

**Figure 3:**
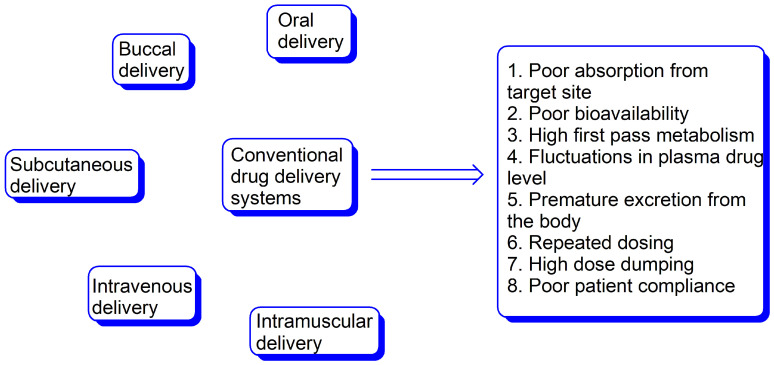
Limitations of conventional drug delivery systems.

Conventional modes of drug delivery suffer from a number of limitations (Figure 3), such as poor bioavailability, resulting in a need for high doses and repeated administration of drugs to stimulate a therapeutic effect, as was observed with opioids, which are mainly orally administered [4,21]. This, however, results in low overall efficacy, poor patient compliance, and side effects because of the fluctuating drug concentration [12]. Transdermal and parenteral pathways are alternatives for delivery. Opioid patches relieve pain for up to 72 h, depending on the physicochemical properties of the drug [21].

**Figure 4:**
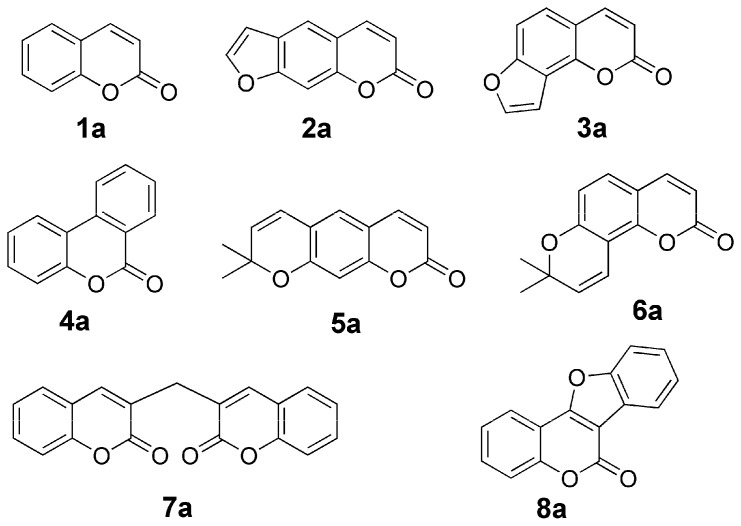
Examples of coumarin derivatives **1a**–**8a** [28].

An illustrative example is represented by coumarin (**1a**) and its derivatives (Figure 4), which demonstrate various types of biological activities, being able to serve as antimicrobial agents, antioxidants, anti-inflammatories, anticancer agents, anticoagulants, antiviral agents [28]. Coumarin, however, suffers from poor bioavailability; once absorbed, only 2–6% reaches systemic circulation as it is rapidly metabolized into 7-hydroxycoumarin (phase I metabolite) and 7-hydroxycoumarin glucuronide (phase II metabolite, suggesting an extensive first-pass effect) [29,30]. Coumarin and its metabolites are excreted via the kidneys, with 7-hydroxycoumarin glucuronide (approximately 60% of the ingested dose of coumarin) being the major metabolite [30]. Although coumarin has been applied as a treatment for lymphedema due to its anti-edematous effect, due to having a short half-life of 1 h, several cases of drug-related hepatitis have been reported because of the overdoses of coumarin. Thus, it has been withdrawn from the market except for the brands with low concentrations of coumarin [31].

There are several key physicochemical properties contributing to the low bioavailability of drugs, as described by Lipinski’s “rule of 5” [3,22,32,33]. The empirical Lipinski’s rule of 5 states that poor absorption of an orally administered drug is likely when one or more of the following rules are violated: the drug molecule has more than 5 H-bond donors (the total number of O-H and N-H bonds) or 10 H-bond acceptors (the total number of O- and N-atoms), the molecular weight is greater than 500, and the partition coefficient between n-octanol and water is greater than 5 [32,33]. Thus, drugs with higher molecular weight and poor solubility in water have poor bioavailability when orally administered due to inefficient absorption into the systemic circulation [32]. Too hydrophobic drugs are prone to precipitation and must be modified to improve their dissolution in aqueous fluids [28,29]. On the other hand, excessively hydrophilic drugs are susceptible to rapid elimination and are unable to cross lipid barriers, such as the blood-brain barrier, thus leading to a reduction in drug bioavailability and life span [28].

Various strategies have been and are currently being investigated to improve the bioavailability of poorly absorbed drugs, as it is estimated that between 40% and 70% of new compounds identified in drug discovery are poorly soluble in aqueous media [32]. Numerous promising drug candidates also have a short half-life, like peptides and proteins, with a half-life of minutes to hours. Therefore, such drug candidates are regularly abandoned [34,35]. Poorly absorbed drugs restrain the therapeutic efficacy; therefore, without an efficient delivery formulation, the whole therapeutic process can be rendered useless [4,32]. Consequently, studies are being carried out to enhance drug absorption using different formulation techniques to improve bioavailability, such as colloidal dispersions and prodrug approaches [32,36].

## 3. Carrier and Bioprecursor Prodrugs as an Alternative to Overcome Limitations of Drug Delivery

To overcome poor chemical stability, solubility, lack of site-specificity, extensive drug metabolism, and permeability of barriers in order to increase the bioavailability of drugs, the prodrug approach was invented [34,37,38]. There are carrier and bioprecursor prodrugs that can be administered through the conventional routes of delivery [37,38,39].

**Figure 5:**
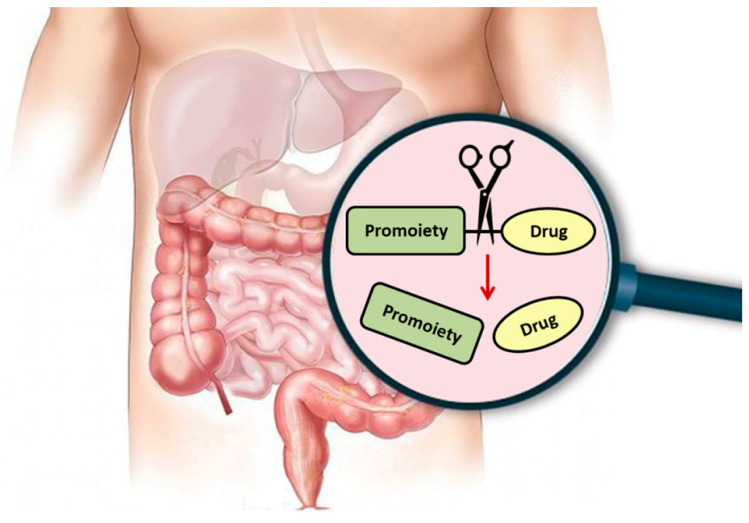
Activation of prodrugs by a cleavage reaction. Reprinted with permission from Ref. [36]. Copyright 2020 MDPI.

Carrier prodrugs are drug molecules conjugated to a promoiety, as shown in Figure 5. The promoiety groups are mostly linked by groups such as esters, anhydrides, carbonates, carbamates, imines, and amides that undergo enzymatic and or chemical transformations in vivo to obtain the active drug and release the promoiety group as a by-product [34,36,37,39,40]. The prodrug approach is used to optimize the bioavailability of new compounds as well as of already marketed drugs [36].

**Scheme 1:**
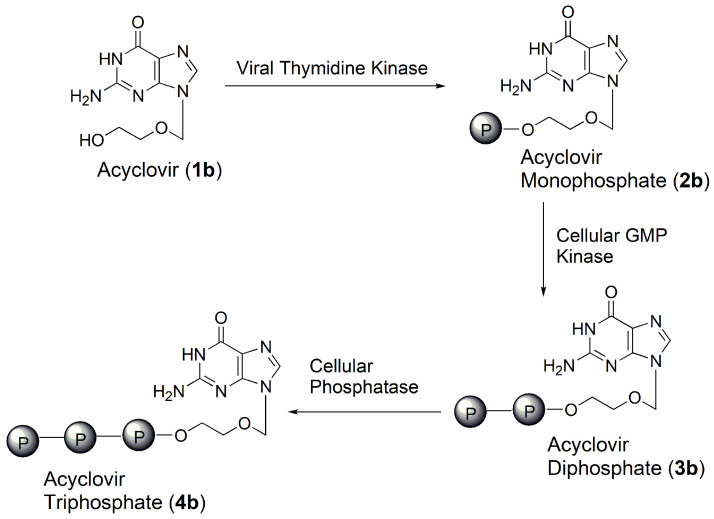
Generation of the Acyclovir active drug in vivo [41].

Bioprecursor prodrugs undergo a transformation in vivo to generate the active drug [39]. For example, Acyclovir (**1b**) is a bioprecursor prodrug. It is a nucleoside analog inhibiting the human herpes virus, including Herpes simplex (VHs) virus, types 1 and 2. Acyclovir undergoes three phosphorylation stages only in infected cells, thus generating an active triphosphate compound [41] (Scheme 1).

**Scheme 2:**
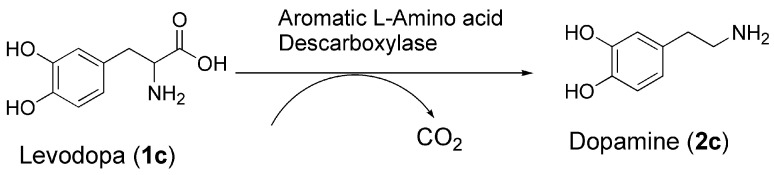
Levodopa as a bioprecursor prodrug for dopamine [41].

Levodopa (**1c**) is also an example of a bioprecursor prodrug for dopamine (Scheme 2). Levodopa has been used to treat Parkinson’s disease, a condition caused by a deficiency in dopamine, which cannot cross the blood–brain barrier due to insufficient lipophilicity [41]. Levodopa is more polar than dopamine and has an amino acid character, which enables transporters of neutral amino acids (LAT1) present in the body to transport it across the cellular membrane, avoiding the blood–brain barrier [41]. Levodopa subsequently undergoes decarboxylation through the action of the aromatic L-amino acid decarboxylase, thus releasing dopamine [41].

**Scheme 3:**
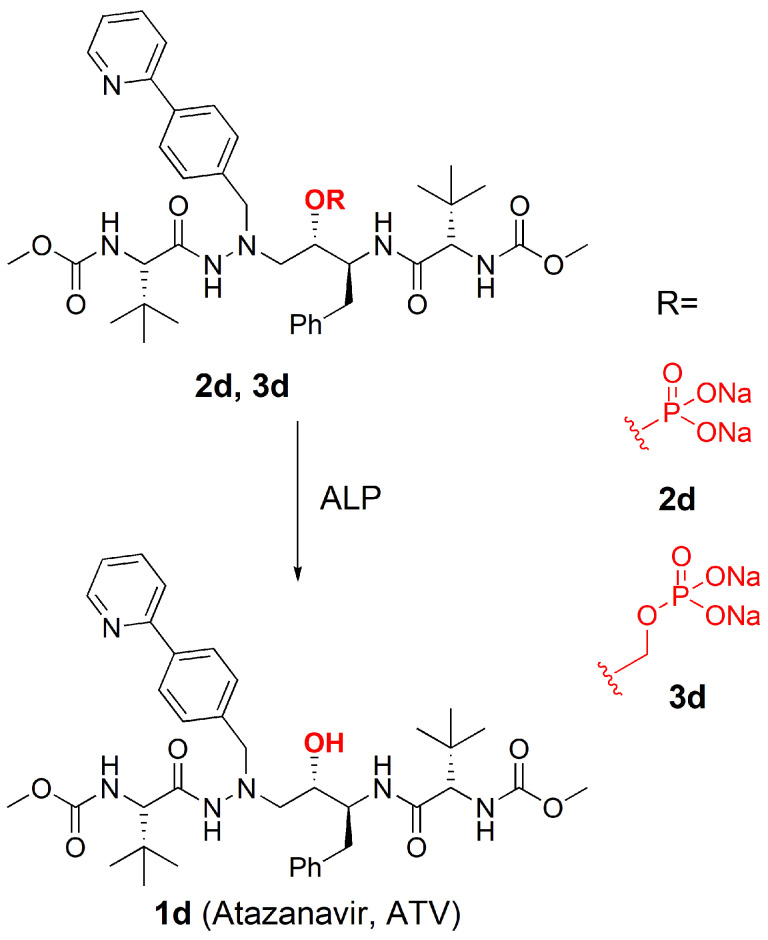
Carrier phosphate prodrugs for atazanavir [39].

It is estimated that approximately 10% of all commercialized drugs worldwide are prodrugs [36,38,42]. However, prodrug activation occurs through uncontrollable enzymatic and chemical transformations, which sometimes fail to release the active drug, while the nature of enzymatic transformations is not fully understood [42]. Prodrugs can also pose challenges for bioactivation in the case where the promoiety group is shielded by steric hindrance, thus making it difficult for the activating enzymes to access and cleave the promoiety group from the active drug [39]. For example, the phosphate ester prodrugs of atazanavir (**1d**) failed to show significant systemic exposure of their respective parent compounds following oral administration to rats [39] (Scheme 3).

**Scheme 4:**
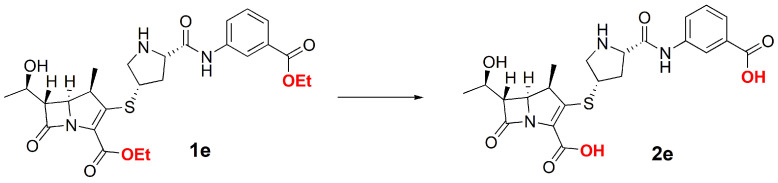
Two activation steps for the release of **2e** [39].

Prodrugs, which require two successive bioactivation steps to release the active drug, often present challenges. As an example, in the case of **1e**, the first activation was fast, whereas the second activation with the release of **2e** was slow, which resulted in the systemic circulation of two different drug species (Scheme 4). Such a scenario will complicate the pharmacokinetic (PK) profile of the prodrug [39].

**Scheme 5:**
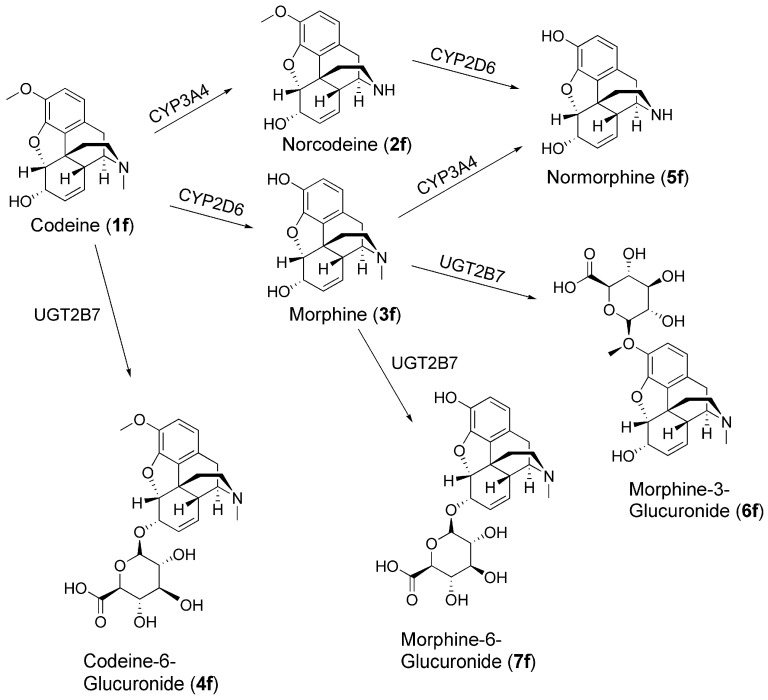
Metabolic pathways of codeine [43].

Codeine (**1f**) is an example of a carrier prodrug that is metabolized to three primary metabolites [43], as shown in Scheme 5. The major metabolic pathway is glucuronidation, while the two oxidative pathways are meditated by the cytochrome P450 enzyme system, namely *O*-demethylation to morphine by CYP2D6 and *N*-demethylation to norcodeine by CYP3A4 [43]. Codeine has a very low affinity for the µ-opioid receptors; therefore, the analgesic effect is from its morphine metabolite [43,44]. Approximately 5–10% of codeine undergoes *O*-demethylation to morphine, with 5% to 15% of codeine being excreted unchanged in the urine [44,45]. However, the full account of codeine metabolism still remains elusive [46].

## 4. Nanotechnology in Smart Drug Delivery

Smart drug delivery systems are capable of overcoming the limitations of conventional drug delivery systems and enhancing the effectiveness and safety of therapeutic drugs [3,4,47]. Smart drug delivery, also known as targeted or intelligent drug delivery, offers control over drug release and reduces the dosage frequency while maintaining drug concentration in targeted organs and tissues for longer periods of time, therefore enhancing the therapeutic efficacy [3,12,35,48,49]. Smart drug delivery allows drugs to be delivered at a controlled rate at a site as precisely as possible to achieve maximum efficacy and safety [4,35,47,50]. Thus, smart drug delivery offers a reduction in drug toxicities and improves the overall therapeutic efficacy [35].

**Figure 6:**
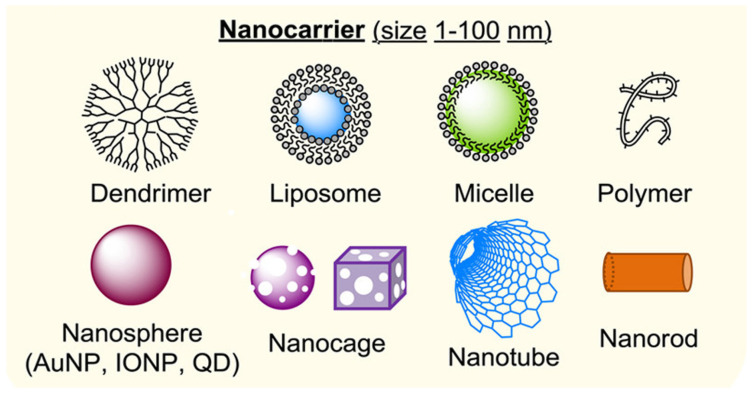
Various structures of nanosized materials. Reprinted with permission from Ref. [56]. Copyright 2015 American Chemical Society.

Smart drug delivery was revolutionized by the application of nanotechnology in the pharmaceutical and medical fields, entailing the use of novel nanosized materials in the medical field, currently referred to as nanomedicine [48,51,52,53,54]. Nanosized structures have huge potential in a number of applications ranging from nanotechnology to biotechnology [55]. Nanotechnology refers to the advanced manufacturing science and engineering of materials at the nanometer scale (1–100 nm) [13,48,52]. Nanosized materials offer a surface area-to-volume ratio over bulk materials and have a high potential for interaction with systems [13,48]. These materials can also take on various shapes, such as globular particles, tubes, and rods (Figure 6), and can function as a nanocarrier and be applied in areas such as drug delivery, tissue engineering, and diagnostic agents [6,13,48,56,57]. Nanomedicine has provided an opportunity to make use of drugs that until now would not be administered and, in general, for the creation of new therapeutics [58]. It is now accepted that nanotechnology will contribute towards a growing number of therapeutic arsenals, with some nanosized drug delivery carriers having been approved for human use already [57,59]. To date, approximately over 100 nanomedicine-based formulations have been approved by the FDA and EMA, with reports suggesting a fast annual increase in the number of nanomedicine-based formulations [57].

## 5. Colloidal Nanocarriers in Drug Delivery

Nanocarriers are commonly colloidal nanoparticles of great versatility that function as drug-delivery vehicles for the transport of drugs or other substances to a target site [6,60]. Nanocarriers should be able to improve the solubility of hydrophobic compounds and render them suitable for parenteral administration. Furthermore, they should increase the stability of therapeutics, such as peptides and oligonucleotides [61]. Colloidal nanocarriers exist in various forms and provide endless opportunities in the area of drug delivery. Therefore, they belong to the area of active research and are being increasingly investigated to harness their potential [61].

Colloid is the term coined by Thomas Graham to describe “pseudosolutions”, the dispersion of one phase into another with a size range of 1 nm to 1 μm. Colloidal systems have often been applied in drug delivery to improve the therapeutic efficacy of drugs [62]. Colloidal drug nanocarriers may be classified as self-assembled lipid systems (emulsions, liposomes, solid lipid nanoparticles, etc.), polymer systems (nanoparticles, micelles, dendrimers, conjugates, etc.), drug nanoparticle systems, and procolloidal systems (self-emulsifying oral delivery systems and liquid crystalline systems) [62,63,64].

### 5.1. Nanocarriers as Organic, Inorganic or Hybrid

**Figure 7:**
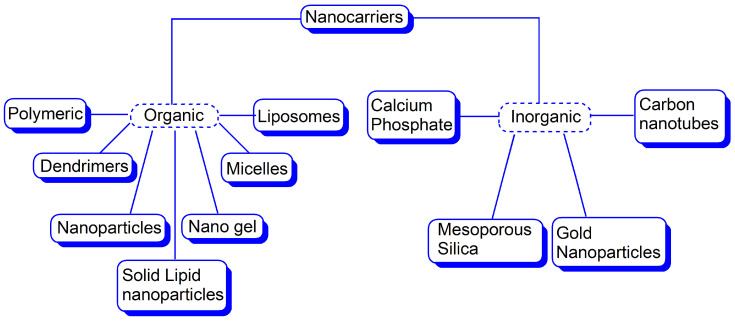
Different categories of nanocarriers.

Nanocarriers can further be described as either organic, inorganic, or hybrid in nature [6,13], as shown in Figure 7. Organic nanocarriers may be polymeric structures, micelles, vesicles, liposomes, dendrimers, solid lipid nanoparticles (SLNs), and nanogels [6]. Inorganic nanocarriers are quantum dots, gold nanoparticles, mesoporous silica nanoparticles (MSNs), calcium phosphate, carbon nanotubes, and similar nanostructures [6,48,65,66].

### 5.2. Organic Nanocarriers

**Figure 8:**
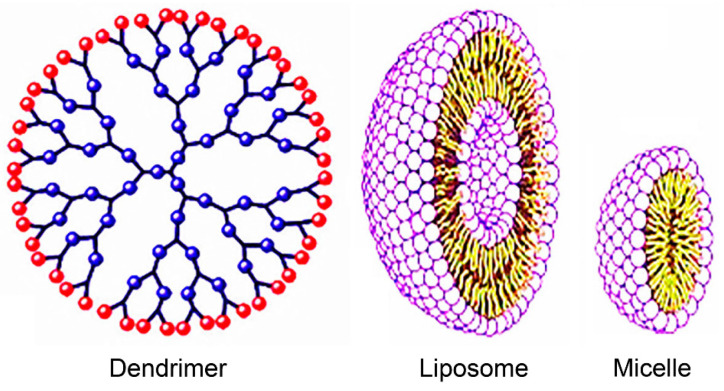
Organic nanocarriers [67].

Organic nanocarriers are versatile systems with the ability to conjugate a variety of drugs as well as ligands for drug delivery [6]. Polymeric nanocarriers and liposome-mediated drug delivery systems represent the first generation of nanocarriers. They are simple molecular systems with liposomes being developed for drug delivery purposes as early as the 1970s [6,64]. Liposomes (also called vesicles) are hollow spherical particles comprised of one or more bilayers, usually made of phospholipids, surrounding an aqueous core (Figure 8) [58,64]. Owing to the presence of hydrophilic and lipophilic domains, they can be used as carriers for both lipophilic and hydrophilic molecules [64]. Liposomes have been investigated for the delivery of vaccines, toxoids, genes, anticancer and anti-HIV drugs [61]. However, liposomes are not extensively applied due to drawbacks, such as low drug-loading efficiency and poor stability [61].

Dendrimers are highly branched polymeric macromolecules with controlled three-dimensional architecture [58,61]. Most dendrimers possess a hydrophobic core and hydrophilic surface; therefore, drug molecules can be loaded both in the interior and on surface groups, depending on their solubility profiles [61]. Due to their binding characteristics, dendrimers are excellent for drug and gene therapy delivery and are reportedly good carriers for chemotherapeutic agents. However, they are rarely applied because most of them are non-degradable under physiological conditions [13,61]. Therefore, toxicity issues warrant extensive research to be carried out on a potential dendrimer nanocarrier to ensure the highest safety before implementation in the medical field [48].

Micelles are defined as colloidal amphiphilic molecules that assemble into nanosized spherical, ellipsoid, cylindrical, or unilamellar structures [64,68,69]. They are composed of amphiphilic monomers in solution, which aggregate and self-assemble at high concentrations (above the “critical micelle concentration”, or CMC), thus forming micelles [68,70]. Micelles have been proven to deliver poorly soluble anticancer drugs due to their hydrophobic core-shell, and they have the ability to deliver the drug at a desired site by enhancing permeability and retention effect [68,70]. However, the stability of micelles in the bloodstream is concerning because critical micelle concentration may change after injection due to the change of the solution ionic strength and intermolecular interactions with other molecular species, and the encapsulated drugs may leak out of the supramolecular assembly [68,70]. Micelles also have a relatively low drug-loading capacity [70].

Nanoparticles (NPs) are attractive in drug delivery as they can increase solubility, enhance drug stability, extend circulation time, and aid transport across biological barriers [71]. Polymeric NPs are biocompatible and biodegradable, which allows their application in drug delivery systems [13]. The main characteristic of the polymeric NP is the controlled release of therapeutic agents [13]. Polymeric NPs are not only used as nanocarriers for pharmaceutical drugs but also to deliver proteins and DNA [13]. Polymeric NPs are especially useful as carriers for hydrophobic small molecules, which may be encapsulated directly within the hydrophobic core of the NPs [71].

### 5.3. Inorganic Nanocarriers

Recently, inorganic nanocarriers, such as mesoporous silica nanoparticles, gold nanoparticles, graphene oxide, and black phosphorus, have shown promising results in drug delivery due to their drug-loading capacity and stability [72]. In contrast to inorganic NPs, organic NPs are widely studied and have outstanding advantages in biocompatibility, but they possess major drawbacks, such as instability and low drug loading capacity [73]. Inorganic nanocarriers are also traceable and, therefore, can be applied in bio-sensing, cell labeling and imaging, and diagnostics [6]. The use of metal NPs in inorganic nanocarriers may result in long-term health issues. As shown by several studies, inorganic nanoparticles, such as ZnO NPs, may lead to oxidative stress and DNA damage [6,73].

### 5.4. Application of Nanocarriers

The application of nanocarriers in drug delivery involves the conjugation of particularly low-soluble drugs with poor absorption profiles to allow their permeability through the biological barriers as well as to offer protection against degradation by proteolytic enzymes, thus improving drug bioavailability [8,11,48,74]. This asset is of great interest, especially for biopharmaceuticals, such as peptide and protein therapeutics, which are commonly short-lived because of protein unfolding and fast enzymatic degradation [75]. Nanocarriers also ensure extended residence time of the drug in the blood, as observed when solid lipid nanoparticles (SLNs) were conjugated to coumarin, which demonstrates activity against methicillin-resistant *S. aureus* (MRSA) [8,11,48,54,76]. MRSA causes endocarditis, chronic osteomyelitis, and pneumonia [76]. SLNs enhance the therapeutic benefits of coumarin against MRSA infections by enhancing the water solubility of coumarin and offering a controlled release pattern with high coumarin concentrations over an extended period of time [76].

Solid lipid nanoparticles (SLNs) are comparatively stable colloidal carrier systems and exhibit the least toxicity due to their biodegradable nature [61]. They have higher drug loading efficiency and render the drug more stable in their lipid matrix, as well as provide a controlled release lasting up to several weeks [61].

**Figure 9:**
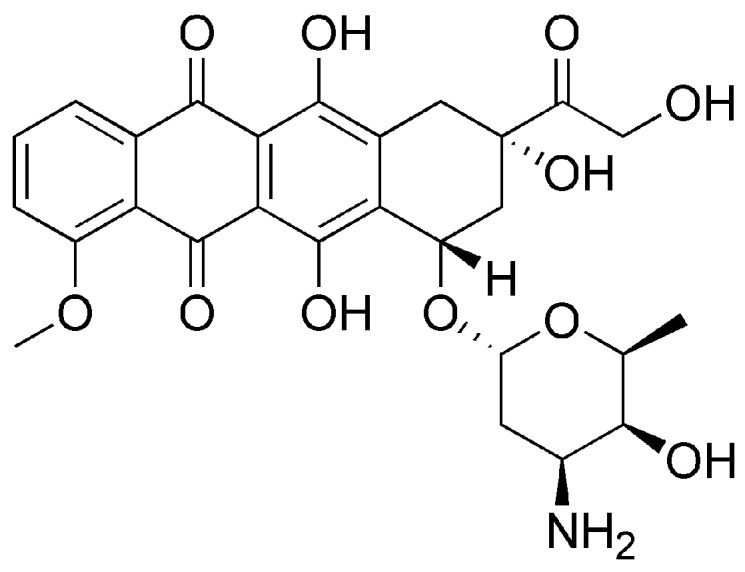
Doxorubicin (DOX), commonly used as an anti-cancer drug.

Anthracycline antibiotic doxorubicin (DOX, Figure 9) is a well-established anticancer drug applied in various chemotherapeutic regimens [62,77,78]. Unfortunately, free DOX has shortcomings of rapid clearance time, resulting in low bioavailability, thus requiring high doses, which limit its clinical use due to its accumulation in the heart, causing cardiomyopathy, which may lead to congestive heart failure and even death [77,78,79]. An approach to reduce the toxicity associated with DOX and enhance its therapeutic efficacy is to use drug nanocarriers [78,79].

Extensive research has been conducted on the conjugation of DOX with various colloidal systems, such as PEGylated liposomes, polymeric micelles, solid lipid nanoparticles, polymeric NPs, dendrimer complexes attached to liposomes, doxorubicin–PEG–folate conjugates and, finally, to tumor-specific antibody-tagged liposomes, for the targeted delivery [62]. Liposomal formulations of DOX, namely Doxil®1, and MyocetTM, which are PEGylated and non-PEGylated liposomal DOX formulations, respectively, have been approved for clinical use and are currently on the market [78,80,81]. Several polymeric NPs loading DOX molecules are currently being evaluated in clinical trials [78]. The major aims of these studies were to enhance the drug solubility and prolong the residence time in the body, provide controlled and stimuli-responsive release, and, preferentially, tumor-specific targeting of the drug [62]. Targeted liposomes are more effective at delivering DOX to the tumor with Lyso-thermosensitive liposomal DOX (LTLD), MM-302, and C225-immunoliposomal(IL)-DOX having reached clinical trials [80].

### 5.5. Delivery of Drugs Through Nanocarriers

**Figure 10:**
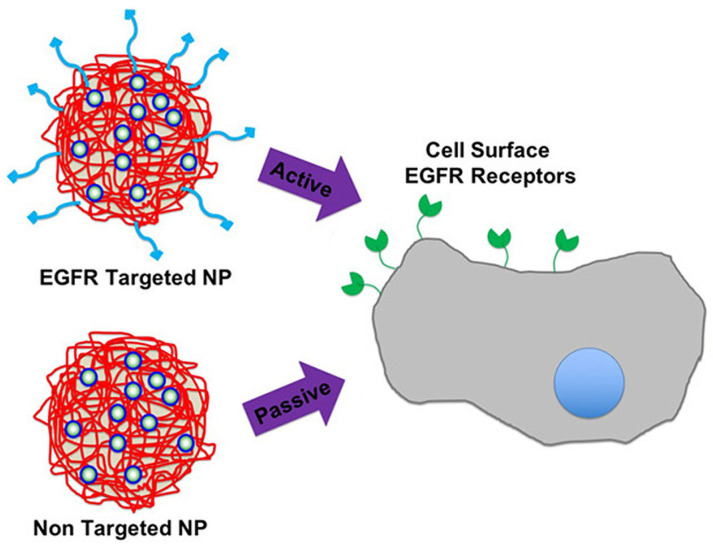
Passive and targeted drug delivery of nanocarriers. The targeted drug delivery nanocarrier has residues complementary to the binding sites on the targeted cell surface. Reprinted with permission from Ref. [84]. Copyright 2018 American Chemical Society.

The delivery of drugs by nanocarriers can be achieved either through passive targeting or targeted delivery [56], as shown in Figure 10. Targeted delivery relies on the attachment of ligands on the nanocarrier surface that bind to specific cell surface biomarkers or receptors [54,56]. They are capable of providing a specific delivery of chemotherapeutic drugs to cancer cells, sparing the normal cells from their cytotoxic effects [73]. Passive targeting occurs when the nanocarrier passively reaches the target organ due to physicochemical, pathophysiological, and anatomical attributes [82]. This occurs more especially in cancerous cells where the tissues have leaky vasculature and poor lymphatic drainage, i.e., enhanced permeability and retention (EPR) effect, resulting in the accumulation of nanoparticles at the tumor site [7,82,83]. The large gaps between adjacent endothelial cells in tumor neovasculature allow for passive targeting of the tumor site, while poor lymphatic drainage leads to enhanced retention of macromolecular therapeutics within the tumor mass [82].

### 5.6. Release of Conjugated Drugs from Nanocarriers

**Figure 11:**
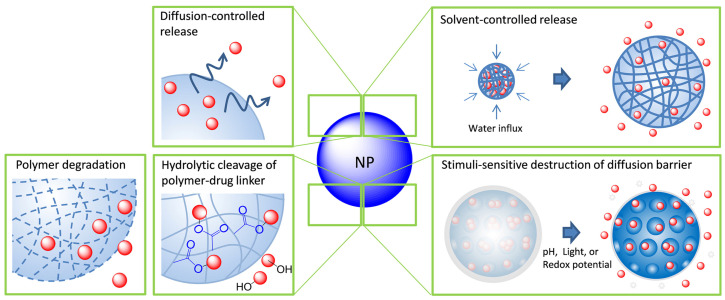
Factors contributing to the release of drugs from nanocarriers. Reprinted with permission from Ref. [85]. Copyright 2015 Elsevier.

The release of conjugated drugs from nanocarriers is influenced by the physical and chemical interaction between the drug, nanocarriers, and additives [85]. The drug can be released through diffusion, solvent (water) dissolution, chemical reaction, and a stimuli response [85], as shown in Figure 11. Release through diffusion is driven by differences in concentration across the membrane, causing the drug to disperse through the polymer matrix [85]. The release by solvent happens more specifically when hydrophilic polymers are placed in an aqueous environment, resulting in an uptake of water, which causes swelling of the polymeric particles and drug release [85]. Chemical reactions resulting in the release of the drug, such as enzymatic or hydrolytic cleavages of ester or amide bonds, are more common for natural biodegradable polymers, for example, poly(amino acids) and polysaccharides [8,85]. Specially designed stimuli-responsive nanocarriers may respond to endogenous stimuli, such as pH, enzyme, glucose, or redox gradient, and to exogenous stimuli, such as temperature and electric field, thus triggering or enhancing the release of the loaded drug [3,51,85]. Often, more than one mechanism contributes to drug release from nanocarriers, although one is designed to be more influential than the others [85].

**Figure 12:**
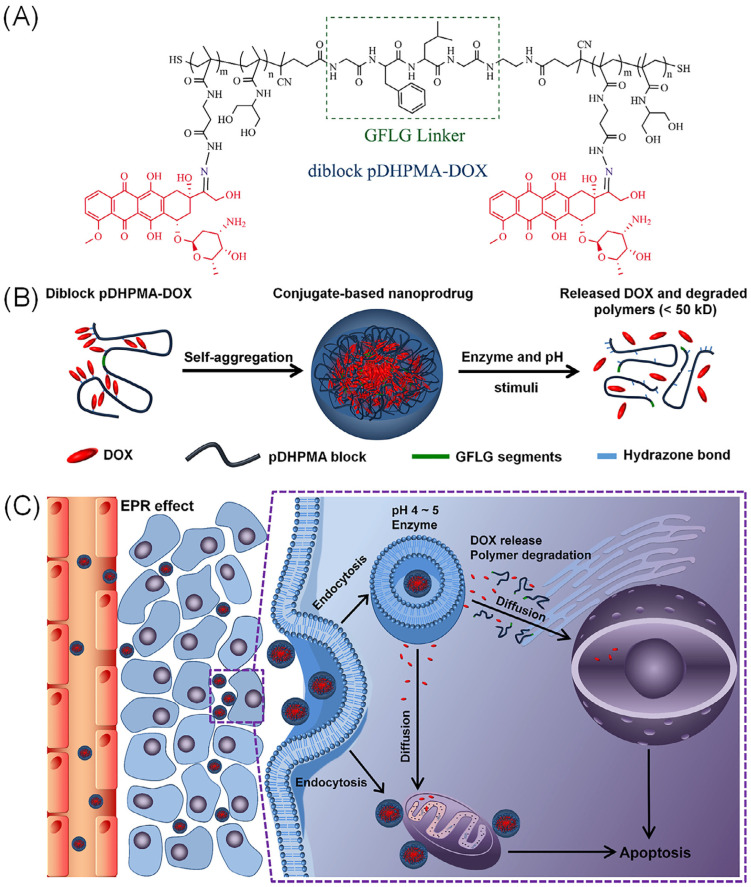
N-(1,3-dihydroxypropan-2-yl) methacrylamide (DHPMA) conjugated with DOX. (**A**) Molecular structure; (**B**) nanocarrier self-assembly; (**C**) cellular function. Reprinted with permission from Ref. [77]. Copyright 2019 Elsevier.

Doxorubicin (DOX) conjugated to N-(1,3-dihydroxypropan-2-yl) methacrylamide (DHPMA) polymer represents a self-aggregation-induced nanoprodrug [77] (Figure 12). The same corresponds to the PEGylated DOX prodrug, which self-assembles into biodegradable micelles [78]. Both DOX prodrugs are pH-responsive due to the hydrazone bond holding these molecular conjugates together. They are split by endocytosis, releasing the active component, DOX, intracellularly, which then induces apoptosis of cancer cells mainly through the mitochondrial pathway [77,78]. Thus, the tumor microenvironmental pH variations induce the targeted release of DOX from the nanoprodrug conjugates [77].

### 5.7. The Effectiveness of Nanocarriers

The primary rationale for using a suitable drug delivery system is its ability to ensure a higher and longer duration of drug bioavailability [48]. The efficacy of smart drug delivery systems depends on the size, shape, hydrophobicity, surface parameters, and several other chemical and physical features of the drugs to be loaded [48]. Ideally, nano-scale materials with high biocompatibility and biodegradability are considered excellent drug delivery systems for biomedical applications [48]. The ideal smart drug delivery system should be inert, biocompatible, mechanically strong, comfortable for the patient, capable of achieving high drug loading, safe from accidental release, simple to administer and remove, and easy to fabricate and sterilize, and, last but not least, it should remain stable in the blood [50,85]. The drug delivery system must ensure efficient delivery of the drug to the targeted cells while preserving the drug’s molecular bioactivity, as well as allow for the control of the kinetics of the drug loading and drug release [73].

## 6. Hydrogel-Based Nanocarriers

Gels are semi-rigid colloidal or polymer networks that expand their volume through a fluid. Organogels are gels prepared in organic solvents, such as hexane, isopropanol, and sunflower oil, while hydrogels are gels prepared in water [86,87]. Interest has been shown towards hydrogels as opposed to organogels, as indicated by the number of related publications with ca. 2250 publications related to organogels during the period 2012–2021 and ca. 71,000 for hydrogels during the same period (data from Scopus) [86]. Such a difference is not surprising since hydrogels can potentially be found in a number of applications within different branches of industry than organogels.

There are over 30 injectable hydrogel products approved by the European Medicines Agency and the US Food and Drug Administration [88]. Due to their appealing properties, they have been applied in many branches of medicine, including cardiology, oncology, immunology, wound healing, and pain management [89,90]. Hydrogels have also been extensively studied in areas such as catalysis, pollutant removal, and energy storage [91].

### 6.1. Composition of Hydrogels

**Figure 13:**
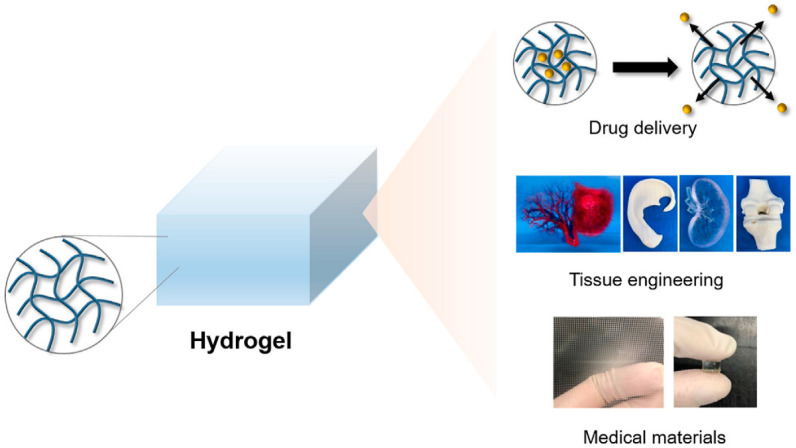
Three-dimensional network in hydrogels and their applications in medicine. Reprinted with permission from Ref. [92]. Copyright 2021 Frontiers.

Hydrogels are a class of soft materials consisting of a three-dimensional (3D) network of hydrophilic polymers crosslinked by covalent bonds (chemical gels) or weak intermolecular interactions (physical gels) [92,93], as shown in Figure 13. The 3D network forms mesh-like structures that afford hydrogels their rich porosity character, with pores commonly filled by water molecules but which can also serve as a depot for the loading of guest molecules, such as drugs [11,75,94,95,96]. These features dictate their application in drug delivery [90]. Hydrogels differ in mesh sizes and architecture of the molecular framework [90]. The hydrophilic nature of hydrogels is due to the presence of polar groups, such as OH, SO_3_H, COOH, NH_2_, and CONH_2_, which enable hydrogels to absorb large quantities of water, resulting in swelling via diffusion [91,97,98,99]. They retain up to 99.5 (wt/vol)% of water, forming a viscoelastic structure that resists dissolution due to the cross-links between the network, thus making hydrogels solid-like but still insoluble [11,21,34,90,93,100,101,102,103]. The crosslinks limit the swelling of the hydrogel in an aqueous environment [104].

The viscoelastic character of hydrogels makes them deformable and able to conform to the shape of the surface to which they are applied [95]. These materials are flexible and soft due to their water-rich character [105]. The hydrophilic groups of the hydrogel not only allow for water absorption but also enable interaction with tissues within the body, such as epithelial tissues and mucous membranes [100]. The water-holding capacity of hydrogels promotes excellent biocompatibility, serves as a great platform for encapsulating drug molecules, and provides physical similarities to tissues and physiochemical similarities to the extracellular matrix [11,90,95,102,105]. The gel network usually restricts the penetration of different enzymes, thus protecting the encapsulated drug from enzymatic degradation and rapid clearance. It leads to the improvement of the general effectiveness of drugs and leads to the reduction of their dosages [11,88].

### 6.2. Chemical and Physical Hydrogels

**Figure 14:**
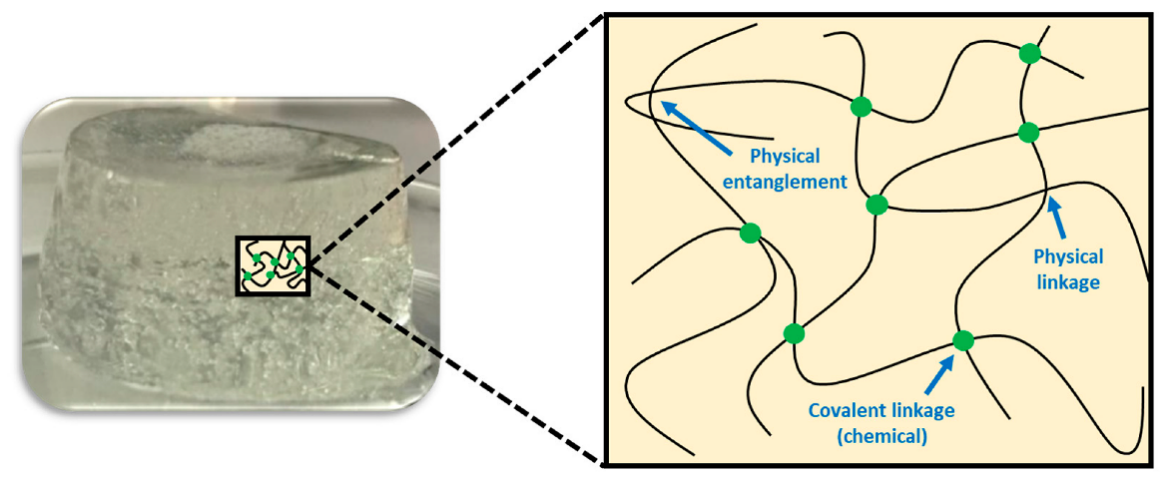
Cross-linking of polymers in gels. Reprinted with permission from Ref. [109]. Copyright 2021 MDPI.

Hydrogels are classified into two major classes: chemical or physical hydrogels [11,12,24,48,93,103], as shown in Figure 14. Chemical hydrogels consist of molecular networks held together by permanent covalent bonds, while physical hydrogels consist of reversible networks held together by non-covalent interactions such as H-bonding, electrostatic, π-π stackings, and hydrophobic interactions [12,21,93,103]. Such molecular networks are interwoven with each other and stabilized by the cross-links (either covalent in chemical gels or based on weak interactions in physical gels) between molecular chains. Physical hydrogels are responsive to stimuli in the local environment, such as temperature, pH, stress, and the presence of specific solutes [12]. Initially, research focused on chemically cross-linked hydrogels from synthetic polymers such as poly(2-hydroxyethyl methacrylate) (PHEMA), poly(3-hydroxypropyl methacrylate) (PHPMA), and poly(hydroxyalkyl methacrylate) [106,107]. In the 1960s, Wichterle and Lim applied poly 2-hydroxethyl methacrylate (PHEMA) biomaterial to contact lenses, and it was the first reported polymeric hydrogel to be used for medical applications [100,108]. In the 1970s, the focus shifted to physical hydrogels, such as PEG-polyester copolymers and poly(N-isopropylacrylamide) (pNIPAAm), which are cross-linked via hydrophobic interactions [106].

### 6.3. High and Low Molecular Weight Hydrogels

Hydrogels are further classified as either low or high molecular weight [102,103]. Low molecular weight (LMW) gelators have a molecular weight below 1000 Da [110,111]. For example, for a peptide-based hydrogel, it means a range of 1 to 20 amino acids [112]. LMW gelators are said to be metastable and often crystallize during prolonged storage, while high molecular weight (HMW) gelators form stable gels that do not crystallize even after several years [113]. In general, HMW hydrogels (usually polymeric hydrogels) tend to release encapsulated drugs faster than LMW hydrogels [112]. However, HMW hydrogels also have good mechanical properties (strength) compared to LMW hydrogels due to the long polymer chain comprising their structures, thus resulting in HMW hydrogels exhibiting high stiffness [114,115,116,117]. LMW hydrogels also suffer from low drug loading capacity and insufficient drug release, which influences their therapeutic efficacy [118,119].

### 6.4. Low Molecular Weight Hydrogels

Irrespective of the limitations associated with LMW hydrogels, interest has been shown in them due to their versatile nature and their ability to assemble and form various entangled aggregates via non-covalent interactions [117,119]. LMW hydrogels demonstrate different morphologies like fibers, rods, ribbons, and nanotubes and are used to construct attractive tools for biomedical applications [117,120,121,122]. Many LMW hydrogels mimic biological systems, therefore, have been employed in drug delivery, tissue engineering, cell culture, wound healing, and biofabrication due to their diversity, ease of preparation, biocompatibility, and low toxicity [119,123].

The mechanical properties of hydrogels are critical for their practical applications [124]. Therefore, attempts have been made to stabilize and improve the mechanical strength of LMW hydrogels through a number of methods, such as preparing nanocomposite hydrogel materials, double-network hydrogel, and dual-cross-linking hydrogel [125]. Co-assembly was utilized, as it was observed when acylhydrazone-based hydrogels incorporated bipyridinium units, and it was found to form highly stable hydrogels through donor-acceptor charge transfer interactions with electron-rich naphthyl moieties [119]. Co-assembly was also observed when sodium alginate (SA) was incorporated into fluorenylmethoxycarbonyl diphenylalanine (Fmoc-FF) gels, forming a double-crosslinked hydrogel, which led to better mechanical properties, high stability, and good biocompatibility [126]. A series of pyrene–amino acid conjugate hydrogelators were developed, and it was reported that a combination of oppositely charged derivatives of pyrene-capped amino acids produced mechanically strong hydrogels [116]. Pyrene and fluorenylmethoxycarbonyl functionalized l-lysine (acting as donor) exhibit charge transfer (CT) behavior when mixed with an electron acceptor such as 2,4,7-trinitro-9-fluorenone (TNF) [116]. The incorporation of metal cations has also been found to improve the gelation properties [116].

### 6.5. Polymer-Based Hydrogels

**Figure 15:**
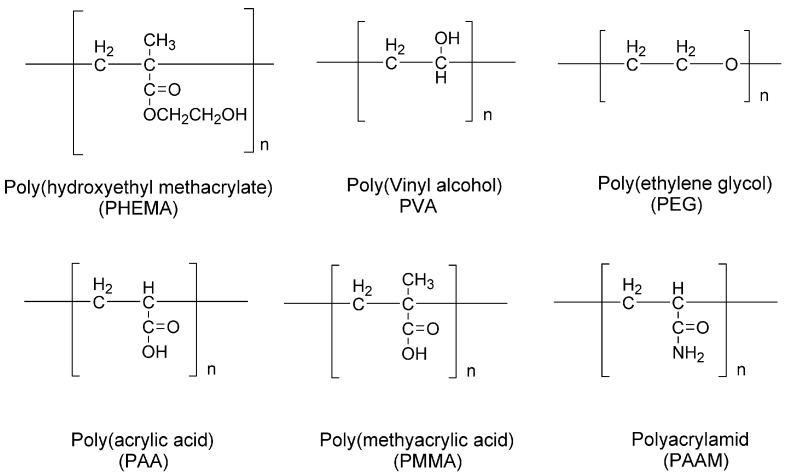
Synthetic polymers used in the preparation of polymeric hydrogels [130].

There is a great diversity of polymeric materials capable of forming hydrogels from natural, semisynthetic, or fully synthetic sources [92,93]. Their molecular weight is always higher than 1000 DA; therefore, all of them belong to the class of HMW gelators. Synthetic polymers used in the preparation of hydrogels include polyvinyl alcohol (PVA), polyethylene glycol (PEG), poly(ethylene oxide) (PEO), poly(2-hydroxyethyl methacrylate) (PHEMA), polyacrylic acid (PAA), polylactic acid (PLA) and polyacrylamide (PAAm) [100], as shown in Figure 15. Synthetic polymers form hydrogels that are mechanically robust and resistant to biodegradation but, however, not biocompatible. Therefore, they require modifications such as PEGylation to enhance their biocompatibility, except for a few, such as polyacrylic acid, which is naturally biocompatible [34,105,127]. Since then, biocompatible and biodegradable polymers have been extensively studied for biomedical applications, in particular, for the fabrication of drug delivery systems [128]. In particular, immense progress has been achieved in the development of polymeric nanocarriers in the form of hydrogels and liposomes [13,129].

Natural polymers are derived from natural sources, exhibit a large diversity of structures, and offer a variety of potential applications [131]. Natural polymers used for hydrogel fabrication include polysaccharides, polynucleotides, proteins, and polypeptides [34,100,102,123]. They are biocompatible, biodegradable, and non-toxic, and, thus, attractive for biomedical applications [34,102,123,125,132]. Peptide-based hydrogels have received much attention due to their ability to mimic natural proteins, particularly extracellular matrix proteins [132]. Although hydrogels based on natural polymers possess attractive features, these materials suffer from several limitations, such as poor mechanical strength [125]. Hence, various strategies have been designed to improve the mechanical strength of the hydrogels [125].

### 6.6. Loading of Drugs into a Hydrogel

**Figure 16:**
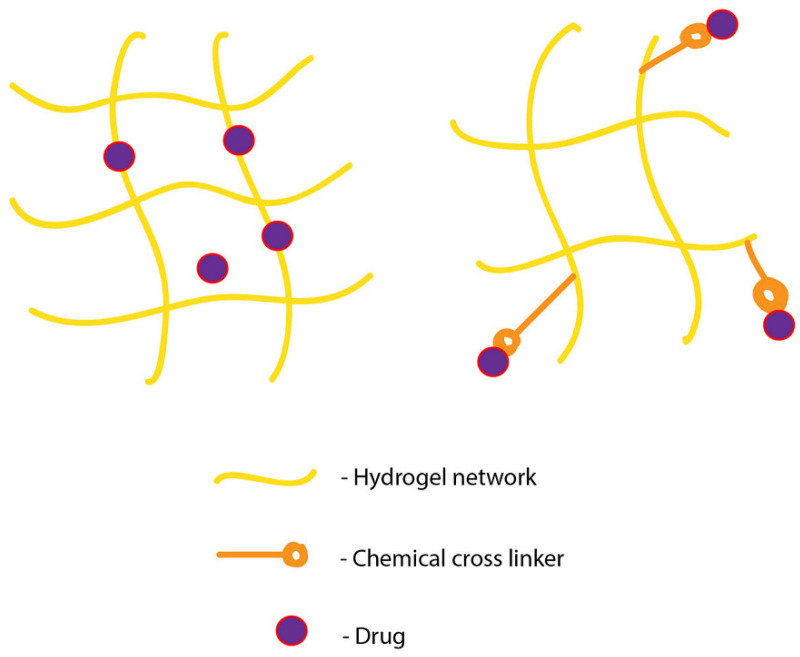
Drugs physically and chemically loaded within a hydrogel. Reprinted with permission from Ref. [13]. Copyright 2019 Springer Nature.

Approaches for loading drugs within a hydrogel include non-covalent immobilization and covalent bonding [95,133], as shown in Figure 16. Diffusion involves the adsorption of drugs onto the pores by soaking the pre-formed hydrogel in a drug-containing solution [133]. The drug diffuses into the pores; the effectiveness of the process is dependent on the structure of the pore, the size of the drug, and its chemical nature [133]. The mesh size of the gel network has to be comparable to the size of the drugs being loaded [11]. This approach is most suitable for small molecules that can easily migrate through the relatively small pores of the hydrogel [133]. The mixing method, also referred to as molecular imprinting, represents the process of mixing the drug, polymer solution, and an initiator with or without a cross-linker to allow polymerization, in which the drug will be encapsulated into the mesh of the hydrogel, usually through non-covalent interactions physically trapping the drug within the hydrogel pores [112,133,134]. This method is ideal for loading larger and hydrophobic drugs [112]. Rarely, drugs may also be covalently linked to hydrogels in the presence of cross-linking agents to form covalent bonds [90,95,135].

### 6.7. Interaction Between Loaded Drug and Polymer Chain

**Figure 17:**
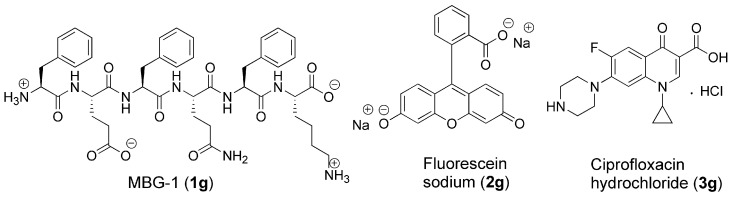
High affinity of MG-1 towards CIP·HCl [137].

Chemical recognition exists through interactions between the polymer chain and the loaded drug with weak intermolecular interactions, such as hydrogen bonding or dispersion forces, creating a balance between strength and responsivity, while much stronger covalent bonds have a poor balance between strength and responsivity, often resulting in poor drug delivery due to the non-responsivity to stimuli [90,112]. The weak intermolecular interactions are the most common interactions and are more suitable for preserving and for the sustained release of loaded drugs [90,104,112,136]. However, hydrogels need to be stable and inert to maintain the drug’s activity through the storage and transport of both the hydrogel and the drug [90]. The polymer chains can possess numerous sites for interactions with the loaded drugs and can be pre-designed using diverse physical and chemical strategies [90]. Interactions to bind drugs to the polymer chains can, for example, include electrostatic attraction and π-stacking through hydrophobic domains [90]. The effect of hydrophobic associations was observed when CIP-HCl (Ciprofloxacin) formed stronger interactions with the MBG-1(H-Phe-GIu-Phe-Gln-Phe-Lys-OH) peptide hydrogelator due to the presence of hydrophobic domains (phenyl group) on the polymer chain that serve as binding sites [90,137] (Figure 17). These interactions resulted in the slow release of CIP-HCI compared to the more polar and hydrophilic fluorescein (**2g**) [137]. The presence of hydrophobic domains on a polymer chain further enables the encapsulation of hydrophobic drugs into hydrogels because the release of water bound to the hydrophobic domains is energetically favorable [90].

Electrostatic interactions made use of in the example of an anionic hydrogel, such as alginate hydrogels, were used to deliver cationic, heparin-binding growth factors, such as vascular endothelial growth factor used to promote tissue regeneration [90]. Sulfonate functional groups are used to increase electrostatic interactions between alginate and protein drugs to extend the release duration [90]. Macromolecules with charges can form multiple ionic bonds with charged hydrogelators through polyion complexation. In this case, the release of the charged macromolecules takes place through hydrogel degradation [104]. Obviously, drug-binding sites within the polymer chains can influence the rate of drug release [104,131].

### 6.8. Delivery of Drug-Loaded Hydrogels

Hydrogels loaded with drugs can be delivered via surgical implantation, local needle injection, systemic delivery, or intravenous infusion [90]. The choice of hydrogel delivery is based on maximizing the overall efficacy and patient compliance [90]. Hydrogels may also be surgically implanted in the body, which, in this case, results in pain and discomfort for the patient, thus limiting their clinical application [89]. Injectable hydrogels have the benefit of overcoming such drawbacks [89]. Drugs loaded in an injectable hydrogel can be delivered at a site and remain within the hydrogel network until there is a stimulus or specific activator molecules present in the body or at the site to trigger the drug release [135].

**Scheme 6:**
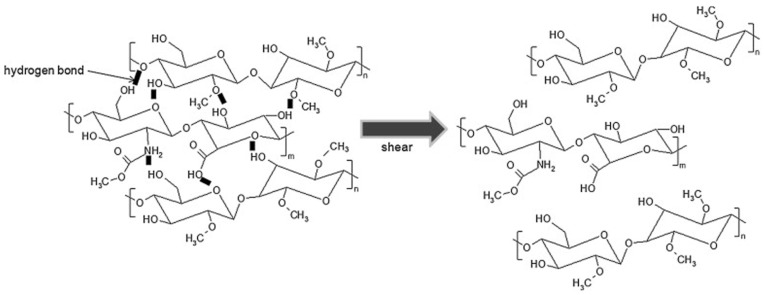
Disassembly and assembly of H-bonding interactions in physical hydrogels are shown as an example of binding between methylcellulose and hyaluronic acid. Reprinted with permission from Ref. [95]. Copyright 2008 Elsevier.

Physical hydrogels are reversible owing to the dynamic competition between pro-assembly weak intermolecular forces (hydrophobic interactions, electrostatic interactions, and hydrogen bonding) and anti-assembly forces (solvation and external mechanical stress) [90]. Physical hydrogels pre-formed outside the body can be injected into a body through the needle using a syringe [90,95]. This is possible because weak non-covalent interactions are reversible, making physical gels deformable under mechanical force, such as shear stress, and enabling their injectability [90,104,109,138]. The application of mechanical force on physical hydrogels through the needle of a syringe results in the disassembly of the non-covalent interactions, causing the hydrogel to shear-thin and exhibit a gel-to-solution transition [75,90,139], as shown in Scheme 6. After removal of the shear stress, a restructuring phase begins, which occurs rapidly as non-covalent interactions reestablish themselves, and the injected mass undergoes a solution-to-gel transition, restoring the initial stiffness of the hydrogel [28,75,90,93].

**Figure 18:**
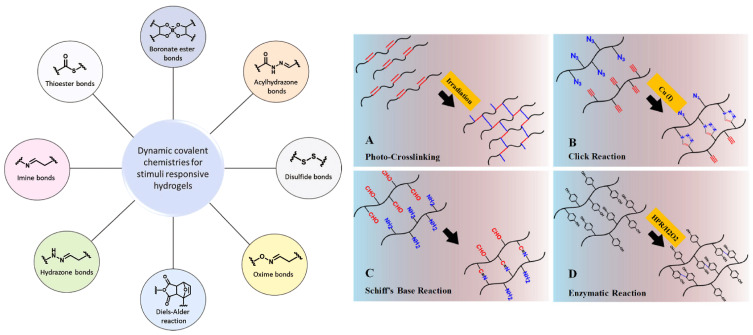
Cross-linking of chemical hydrogels via dynamic covalent bonds. Reprinted with permission from Refs. [97,142]. Copyright 2020 and 2022 MDPI.

Pre-formed chemical hydrogels are not injectable as they are highly stable with a higher mechanical strength due to the permanent linking of covalent bonds, which prevent the dissolution of the polymer chains [114,140]. They require either surgical implantation or an in situ gelling method for delivery [75,92,109]. However, chemical hydrogels containing dynamic covalent bonds are reversible, resulting in self-healing and stimuli-responsiveness, therefore enabling injectability due to the self-healing capability [141,142,143]. Dynamic covalent bonds are capable of associating or dissociating in response to various stimuli. For example, the RS–SR bond can be cleaved by reduction, thiol-disulfide exchange, or light [142,143]. Hydrazones are stable under neutral and basic conditions but readily hydrolyze under acidic conditions and exchange in the presence of hydrazides, aldehydes, or ketones. Several instances of dynamic covalent bonds are shown in Figure 18 [142]. As an example, dextran polymer cross-linked using a dynamic covalent (double Michael addition of thiols to alkynones) impart the dextran hydrogel with shear-thinning and self-healing capabilities, thus enabling hydrogel injection [141].

However, the majority of self-healing hydrogels possess poor mechanical properties. Therefore, efforts to strengthen the mechanical properties of self-healing hydrogels have been reported, e.g., by preparing composite hydrogels or through the increase of hydrophobic interactions [142]. Composite hydrogels involve combining a hydrogel matrix with other materials, such as inorganic fillers, organic compounds, or other polymers, to improve the mechanical properties [144]. Graphene-based composite hydrogels are comprised of graphene nanosheets within a hydrogel matrix, in which graphene improves the mechanical strength, stiffness, and toughness of hydrogels, enabling their utilization in load-bearing applications. Graphene oxide (GO) is the most widely studied inorganic filler [145]. Unfortunately, the use of such inorganic fillers strongly diminishes the biocompatibility of such materials.

**Figure 19:**
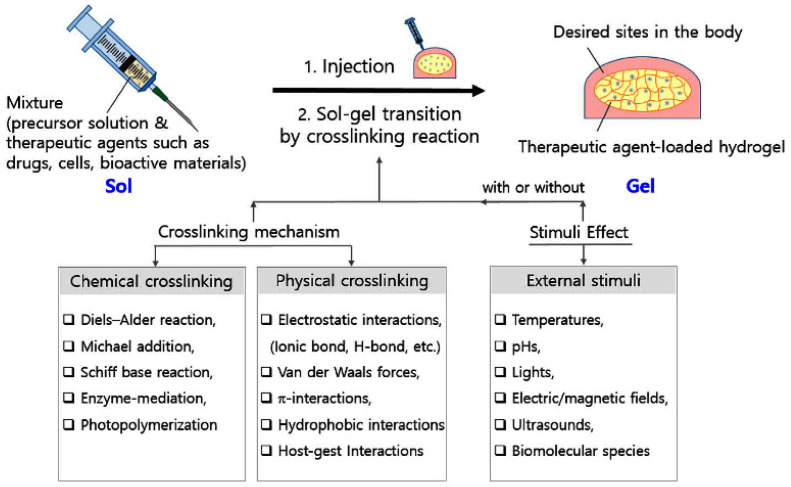
In situ gelation of physical and chemical hydrogels. Reprinted with permission from Ref. [89]. Copyright 2018 Springer Nature.

Injectable chemical hydrogels that undergo gelation in situ have been reported (Figure 19), whereby a mixture of the polymer and therapeutic drug is mixed with an initiator and a cross-linker [146]. The mixture in the syringe is then immediately administrated at a desired site in the body [89,109]. These injectable hydrogels may be prepared in situ using reactions such as the Diels Alder reaction, alkyne–azide click reaction, Michael reaction, Schiff base reaction, photo-mediated reactions, or enzyme-mediated reactions [89,147]. The in situ gelation enables hydrogels to polymerize with the drug inside the matrix [146]. It is essential for hydrogels to undergo solution-to-gel transition at the site of injection in a precisely time-dependent manner when applied in the body to avoid washing out of the drug or individual hydrogel fibers by the body fluids [135]. Injectable chemical hydrogels without dynamic covalent bonds possess higher mechanical strength and longer stability in comparison to physical hydrogels [89,148]. The use of cross-linking agents in injectable chemical hydrogels may limit their application due to the negative effect on biocompatibility; however, recent studies gave access to injectable chemical hydrogels without any toxic cross-linker, although their responses to external stimuli were usually limited [89,148].

### 6.9. Release of Loaded Drugs Through Surface or Bulk Erosion of Hydrogels

**Figure 20:**
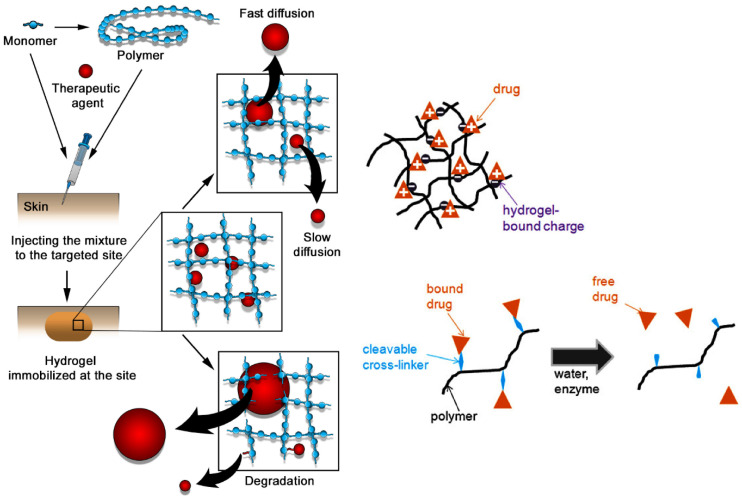
Different release modes of loaded drug from hydrogel. Reprinted with permission from Refs. [91,95]. Copyright 2021 Wiley, 2008 Elsevier.

Drugs loaded within a hydrogel can be liberated to the surroundings by two different release mechanisms: diffusion or hydrogel swelling. In the first one, the mesh size increases, thus releasing drugs; in the second one, degradation of the labile drug-polymer interaction takes place [75,99,146,149], as shown in Figure 20. Release through diffusion is described by Fick’s law as the most common model of drug release [112,146]. The release of loaded drugs is dependent on the mesh size of the matrix, which is also controlled by factors such as the degree of cross-linking, chemical structure of the monomers and intensity of external stimuli, size of the loaded drug, and mechanical properties of the hydrogel [11,12,146]. Hydrogels applied in the biomedical field usually have a mesh size range of 5 to 100 nm, which is, on average, much larger than the molecular sizes of most of the small molecule drugs [146]. When the mesh size is larger than the drug, the most dominant mode of drug release is diffusion, as the small drugs move freely within the network. However, this often results in the fast release rate of the loaded drug [12,90,146]. When the mesh size is smaller than the loaded drug, as is observed for macromolecules like oligonucleotides, peptides, and proteins, the loaded drug remains entrapped within the hydrogel network [146]. Diffusion is suppressed, and the release of the drug is dependent on the degradation of the hydrogel network [90,91,99,146]. The mesh size increases as the network degrades, allowing drugs to sustainably diffuse out of the hydrogel [90]. Degradation is usually meditated by hydrolysis. It can also be triggered by local stimuli, such as acidic conditions, or mediated by enzyme activity. Hydrolysis usually occurs in the polymer backbone or at the cross-links [90,91,131,146]. If drugs are covalently bound to the polymer backbone, the bond is usually labile and can be easily broken by hydrolysis or enzymatic degradation (e.g., of an ester bond) [131].

The rate of hydrogel degradation influences the rate of cargo release and may occur through the bulk or on the surface of the hydrogel and is accompanied by loss of hydrogel mass (Figure 20) [90,91]. In bulk erosion, degradation occurs throughout the hydrogel, which entails mass loss, whereas in surface erosion, degradation is limited to the surface and proceeds via an erosion front [91,150]. Hydrophilic gels are usually completely permeable to water and undergo bulk erosion, while hydrophobic gels may undergo bulk or surface erosion [131].

The majority of hydrogels undergo bulk erosion due to the permeability of the network to water or enzymes, which facilitates degradation. When the rate of water and enzyme diffusion is faster than the rate of bond degradation, degradation occurs simultaneously throughout the bulk of the hydrogel [90,151]. Bulk erosion is homogenous and occurs instantaneously, leading to a loss of mass [152]. Polymers with low molecular weight tend to degrade faster when a large area of the polymer is in contact with water, resulting in an accelerated drug release [151]. Bulk erosion occurs in three phases during drug release: initial drug burst, diffusive phase, and final drug burst [151]. The initial burst is due to drugs distributed on and near the surface of the polymer matrix and depends on the surface area of the hydrogel, the quantity of drugs loaded, and the solubility of the drug [151]. High drug loading and hydrophilic drugs are most likely to result in an intense initial burst, which may be adverse, especially if high concentrations of the loaded drug are cytotoxic [151].

Surface erosion occurs when the rate of bond breakage is more rapid than the rate of enzyme or water diffusion from the exterior into the bulk of the hydrogel network [90,149,151,152]. Cross-linking polymers with hydrophobic groups can inhibit water entry, leading to surface erosion of hydrogels, which is the mode of drug release [90]. Surface erosion is thought to provide more control over drug release than bulk erosion because drug release is regulated mainly by surface degradation of polymers [151]. Degradable hydrogels are particularly advantageous because drug release can be controlled via degradation, and there is no need for hydrogel removal once the payload is depleted [153].

## 7. Peptide-Based Hydrogels

**Figure 21:**
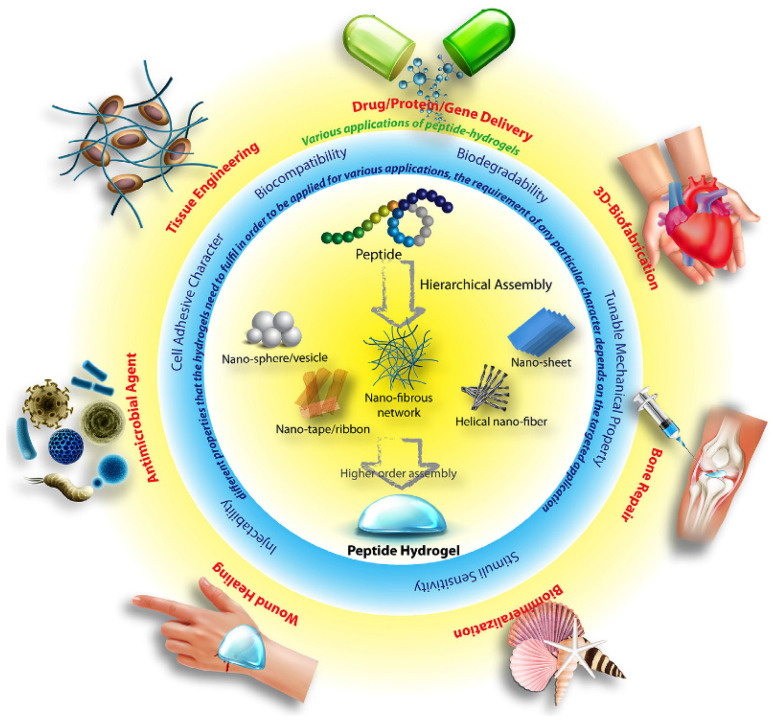
Application of peptide hydrogels. Reprinted with permission from Ref. [135]. Copyright 2021 Frontiers.

Peptide-based hydrogels are a subgroup of natural hydrogels that are particularly attractive due to their diversity in the peptide sequence, biocompatibility, ease of biodegradability, low cytotoxicity, ease of synthesis, and excellent injectability with thixotropic behaviors [21,24,55,75,93,103,132,138,154]. Due to their impressive features, peptide hydrogels have attracted much attention for biomedical applications (Figure 21) [103].

Several peptide hydrogels are commercially available, such as PuraMatrix (Corning, NY, USA), HydroMatrix (Sigma-Aldrich, Burlington, MA, USA), Biogelx (Sigma-Aldrich, Burlington, MA, USA), and PGD-HydroGels (PeptiGelDesign, Cheshire, UK) [140,155]. PuraMatrix, consisting of a 16-amino acid sequence (AcN-RADARADARADAR-CNH_2_), has fiber and pore sizes that are <10 nm and 5–200 nm, respectively, similar to those of the extracellular matrix [156]. PuraMatrix has been applied in cell cultures, such as the nerve, cartilage, liver, cardiomyocyte, and vascular endothelial cells [156].

### 7.1. Natural Amino Acids

**Figure 22:**
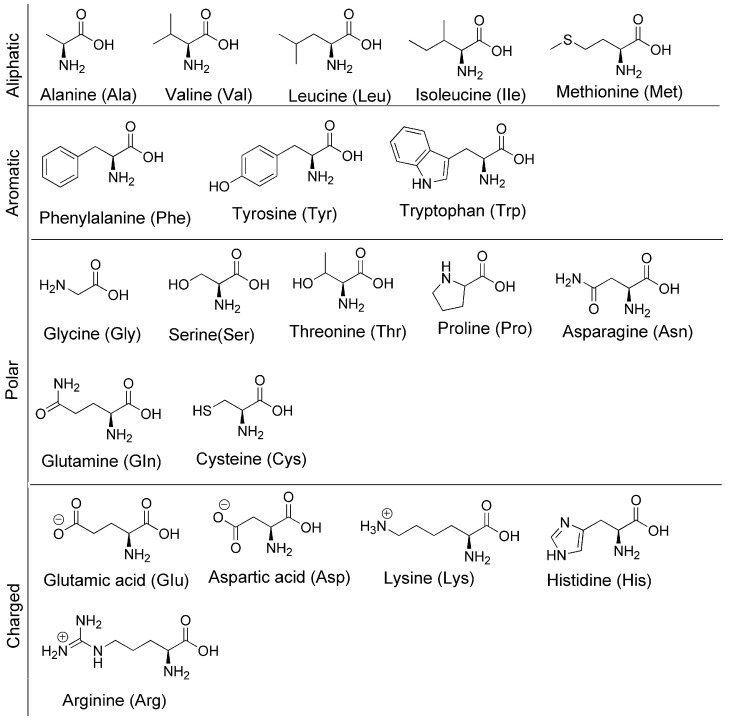
Structures of 20 canonical amino acids.

Peptides are versatile building blocks that form highly structured assemblies that may result in the formation of hydrogels and nanoparticles due to the chemical diversity of amino acids [93,157]. There are 20 canonical α-amino acids that are encoded by the ribosomal machinery for the synthesis of proteins and peptides [158,159]. They represent universal building blocks with different residues appended to the chiral center, as shown in Figure 22 [55,160,161,162]. Protein synthesis relies on the genetic encoding of 20 canonical amino acids, with two non-standard amino acids, which are proteinogenic, Selenocysteine (Sec) and Pyrrolysine (Pyl) [161,162]. Pyl appears only in proteins of Archaea organisms and a few bacterial genera, while Sec is found in all kingdoms of life as the building block of selenoproteins [162,163].

**Figure 23:**
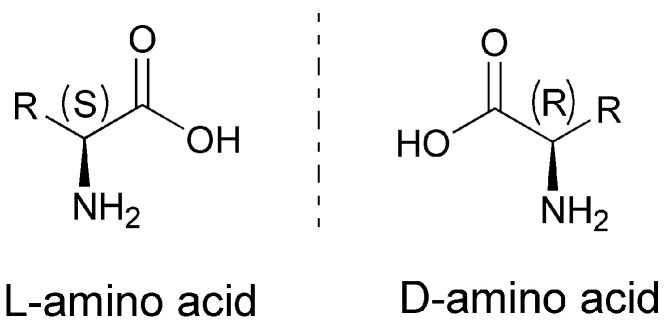
L and D enantiomers of amino acids.

In nature, amino acids exist in two forms of levorotatory (L) and dextrorotatory (D) enantiomers (Figure 23), with different spatial orientations of the four substituents attached to the stereogenic α-carbon [55,164,165]. During evolution, L-AAs became preferred for protein synthesis and main metabolism; hence, they are proteinogenic, while D-AAs, in most cases, are not used for the synthesis of proteins as they are not encoded by the RNA; they are occasionally found in bacterial cell walls, some marine invertebrates, and higher organisms [163,165,166,167,168]. Thus, all canonical amino acids exist as L-forms except for glycine, which is achiral [55,163,169]. All the L-amino acids have the “S” configuration except for cysteine and selenocysteine. L-AAs act as substrates for D-AAs synthesis in the presence of the enzyme racemase, which converts L-AAs to D-AAs [166,168]. The change of L-AAs to D-AAs in a protein leads to a change in its structure and, ultimately, its function and biological activity [165].

### 7.2. Peptide Synthesis

**Scheme 7:**
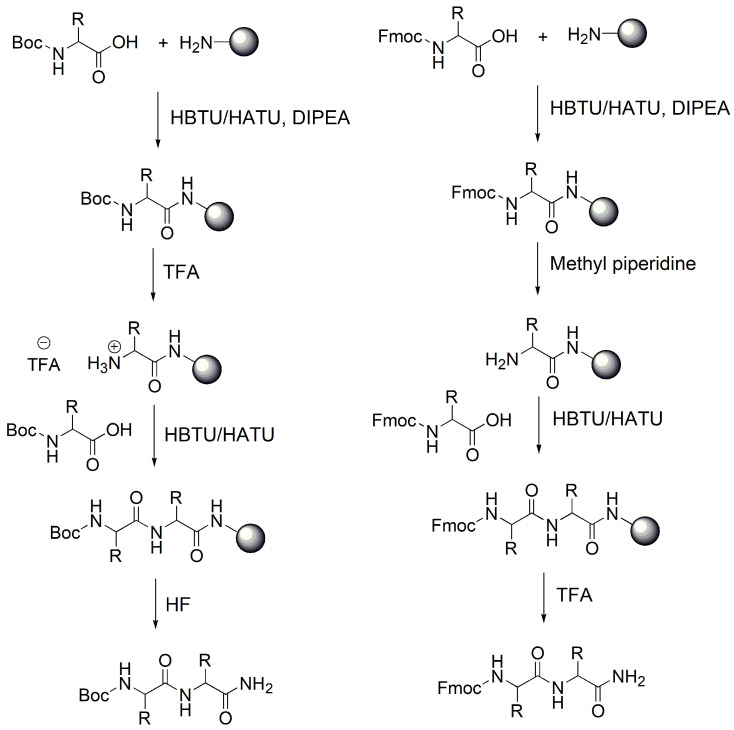
SPPS based on Boc and Fmoc chemistry.

Incorporation of amino acids to form peptides and proteins occurs naturally in living cells. However, they may also be synthesized in laboratories, using precise control over a peptide sequence [123,170,171]. Solid-phase peptide synthesis (SPPS) and solution-phase synthesis (SPS) are two major chemical techniques used for peptide synthesis [172]. During SPPS (Scheme 7), the resin is used to anchor the growing peptide. Firstly, the amino acid is attached to the resin via its C-terminus, with the alpha-amino group being protected to avoid polymerization [172]. After the addition, the protecting group is removed to allow bond formation with the newly added amino acid, which is accompanied by the release of a single water molecule [171,172]. After addition, the process is repeated until the desired peptide is formed, and then the peptide is cleaved from the resin [172]. Boc and Fmoc are the most common protecting groups used and are removed under acidic and basic conditions, respectively [172]. Various resins have been used in SPPS, for example, polystyrene, Merrifield, hydroxymethyl, phenylacetamidomethyl, Wang, Sieber, Rink amide, and 4-methylbenzhydrylamine resins [172,173].

SPPS based on Fmoc chemistry is the most popular choice for synthesizing peptides nowadays [172]. Since the invention of solid-phase synthetic methods by Merrifield in 1963, the number of research focusing on peptide synthesis and their applicability has grown exponentially, finally enabling the routine production of peptides on an industrial scale [172,173].

### 7.3. Folding of Peptides

In an aqueous solution, peptides fold and adopt specific secondary structures through interactions within the peptide backbone and between the side chains, thus self-assembles into well-ordered structures, from nanosheets and nanotubes on the nanoscale to fiber bundles on the macroscale [140,154]. The ability of peptides to fold differently depends on the amino acid sequence and provides an opportunity to design nanoscale materials that are not easily available with traditional organic molecules and polymers [102].

**Figure 24:**
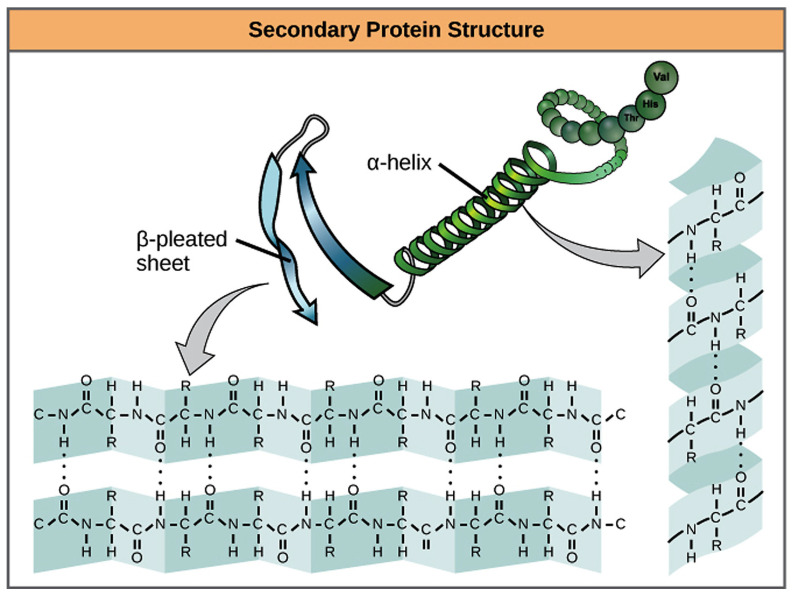
α-helix and β-sheet secondary structures of peptides [175]. Image credit: OpenStax Biology.

The most common secondary peptide structures (first stage of peptide folding) are α-helix and β-sheets (Figure 24), both of which are held together and stabilized by hydrogen bonding within the peptide backbone [140,174,175]. α-Helices have constant displacement distances between nitrogen (N) and alpha carbon (C_α_) of N-C_α_ = 1.47 Å, between C_α_ and carbonyl carbon (C) of C_α_-C = 1.53 Å, and C-N = 1.32 Å [176]. There are 3.6 amino acid (AA) residues per turn, in which the carbonyl group (C=O) of one amino acid forms hydrogen bonds with the fourth amino acids amide group (N-H), resulting in a helical structure [176,177]. The challenge with α-helices is the need for longer peptide sequences to stabilize the helical structure [177]. β-Sheets are formed when adjacent peptide strands line up next to each other, forming a sheet-like structure held together by hydrogen bonds while the side chains extend above and below the plane of the sheet [174,178]. The strands of a β-pleated sheet may be parallel, pointing in the same direction, or antiparallel, pointing in opposite directions [175].

### 7.4. Self-Assembly of Peptides

Self-assembly is very common throughout nature, as observed, for example, in DNA double helix formation and protein folding through secondary, tertiary, and quaternary structures and in the formation of cell membranes upon self-assembly of phospholipids and other amphiphilic molecules [55,139]. Peptide self-assembly is determined by the interplay of thermodynamic and kinetic factors, and the whole process is controlled by the balance of attractive and repulsive forces within and between the peptide molecules [55,102,135,140,154,157,177]. In solution, peptides adopt thermodynamically favorable conformations (e.g., α-helix and β-sheet) in a low-stable energy state due to inter- and intramolecular non-covalent interactions forming ordered nanostructures [179,180]. Thus, the self-assembly of nanostructures is also controlled by thermodynamic parameters, such as pH, temperature, ion concentration, and solvent nature [139]. Self-assembly can be defined as a bottom-up construction where the molecular building blocks interact via weak non-covalent interactions (much less frequently via formation of labile covalent bonds, such as imine bonds) and results in the construction of nanostructures with desired function [154,181]. The study and use of such non-covalent interactions belong to the realm of “supramolecular chemistry”, a name that was coined by Jean-Marie Lehn [181,182].

A plethora of peptide-based hydrogels and proteins adopt β-pleated sheet architecture, while the α-helix architecture is rarely observed in peptide hydrogels [102,135,183,184,185]. β-Sheet peptides are of particular interest as these peptides allow the fabrication of various stable materials in general and hydrogels in particular [186]. Boden and co-workers proposed principles to design peptides of minimal complexities that self-assemble into β-sheet type aggregates in solution: (i) there must be interaction between the side chains (cross-strand attractive forces) through noncovalent interactions; (ii) lateral recognition between the adjacent β-strands to constrain their self-assembly to one dimension and avoid heterogeneous aggregated β-sheet structures; (iii) strong adhesion of water to the surface of the sheets [187]. Boden’s design principle has been applied to create β-sheet forming peptides through the incorporation of repeats of alternating polar and nonpolar amino acids forming hydrophilic and hydrophobic faces, therefore creating an amphiphilic character within the peptide structure that allows higher order assemblies [135]. This family of amphiphilic peptides is known to be capable of self-assembling into antiparallel β-sheet-rich fibers and forming stable hydrogels [138,188]. The self-assembly process of amphiphilic peptides depends on the balance between the hydrophobic and hydrophilic interactions as well as hydrogen bonding [12].

**Figure 25:**
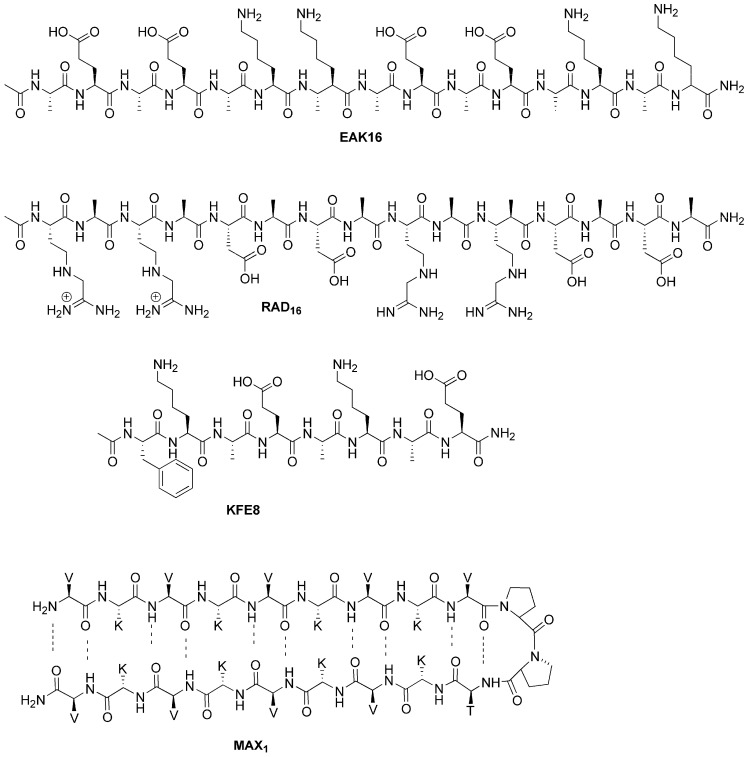
Classical examples of amphipathic peptide hydrogelators [96,190].

Boden’s design principles have been applied to synthesize anionic and cationic peptides, such as RAD16 and EAK16 ionic self-complementary oligopeptides shown in Figure 25. They comprise alternating ionic hydrophilic and hydrophobic amino acids that form stable β-sheet structures in an aqueous media [96]. RADA16 is the first commercially available self-assembling peptide hydrogel designed by Zhang et al. [96]. Multiple amphiphilic peptides that adopt β-sheet secondary structures in aqueous media, such as KFE8(Ac-FKFEFKFE-NH2), MAX1, MAX8, and P^11^, are classical examples of designated amphiphilic peptide hydrogelators [24,93,189]. The beta-hairpin folding peptide MAX1 was developed by Schneider et al. [190].

### 7.5. Hydrogelation of Peptides

**Figure 26:**
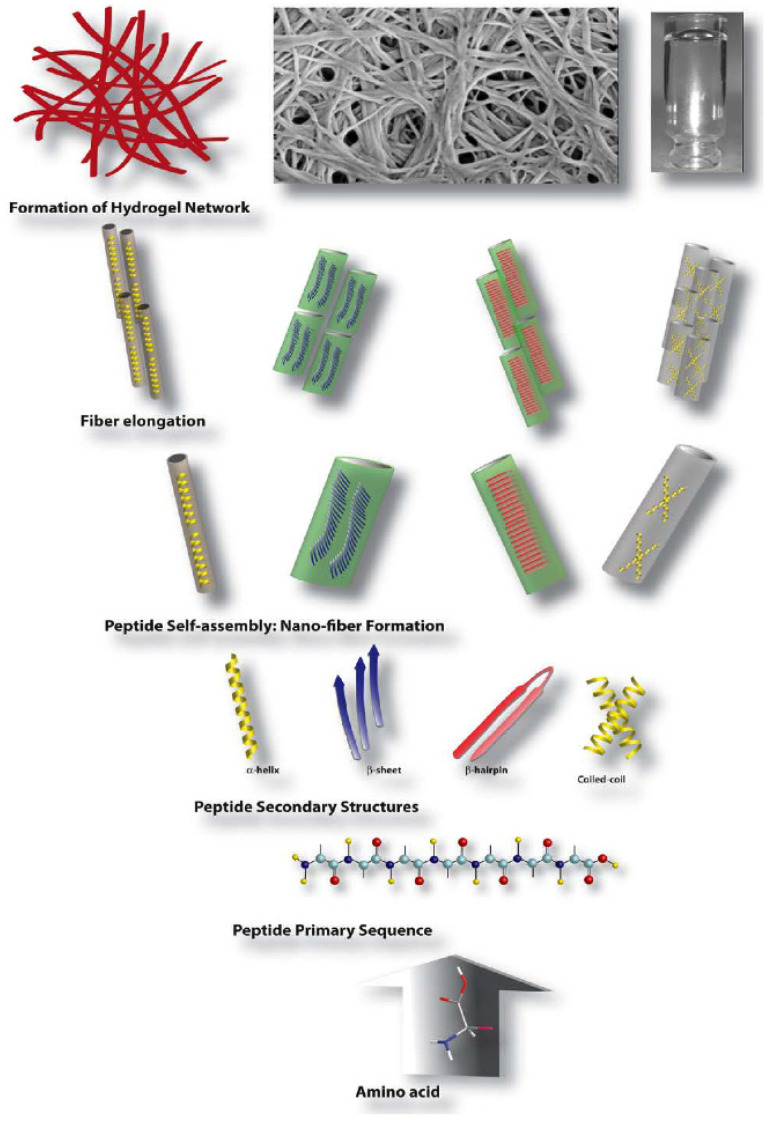
The self-assembly of peptides leads to hydrogelation. Reprinted with permission from Ref. [102]. Copyright 2013 Royal Society of Chemistry.

The order of amino acids in a peptide sequence determines the interaction between the side chains and, thus, influences the secondary structure which the peptides assemble into [53]. This refers to the self-assembly of peptides: the ubiquitous spontaneous organization of peptides into more complex ordered aggregates following a hierarchical path, with peptide hydrogel being at the top of the hierarchy [102,135,154,157,177,179,191], as shown in Figure 26. Therefore, physical peptide hydrogels are dependent on the peptide sequence to lead to secondary structures capable of triggering hydrogelation under favorable environmental conditions, such as pH, ionic strength, and temperature [103,135,153,189,192]. The mechanical strength of peptide hydrogels is influenced by the peptide sequence, supramolecular interactions, pH, temperature, and ionic strength of the gelation medium [132,135]. The strength of hydrogels increases due to the degree of cross-linking: the more cross-links are present, the stiffer the hydrogels and the more brittle they may be [140]. The strength of peptide hydrogels is determined using rheological methods, and rheological properties dictate hydrogel applicability for a particular purpose [135]. Rheology determines the deformation of matter under the influence of imposed stress [193]. Modifications of the peptide sequence or additives in the peptide hydrogel can alter the mechanical strength of peptide hydrogels [135].

Minimalistic dipeptide-based hydrogels are popular; however, they typically require the presence of additional aromatic groups attached to the dipeptide to enhance gel formation as well as gel mechanical strength [116]. This was observed with L-Phe-L-Phe (FF) peptide, which is a popular building block for hydrogel synthesis. However, FF cannot gelate into a hydrogel in the absence of aromatic groups, such as Fmoc, attached to it; otherwise, it forms crystalline nanotubes [94,103]. Fluorenylmethyloxycarbonyl-protected diphenylalanine (Fmoc-FF) developed by Ulijn and colleagues forms a hydrogel. As Smith and coworkers confirmed, Fmoc-Phe-Phe self-assembles into nanocylindrical fibrils based on π-π interlocked antiparallel β-sheets [121,135,184]. The Fmoc group provides the driving force for peptide self-assembly by enhancing intermolecular interactions through an establishment of hydrogen bonding from the carbonyl group and hydrophobic interactions (π–π stacking) through the fluorenyl group [116,177]. The influence of Fmoc was also observed in Fmoc-Phe-DAP hydrogel, which exhibits stable mechanical properties and robust shear-thinning behavior [116].

**Figure 27:**
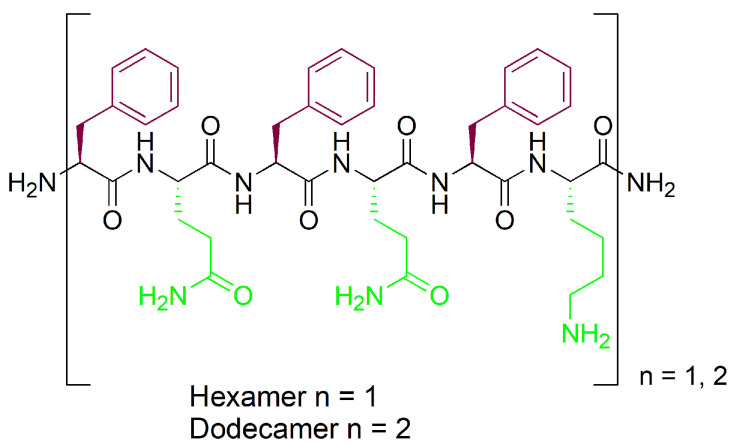
Structure of H-FQFQFK-NH_2_ and its dimer [24].

**Scheme 8:**
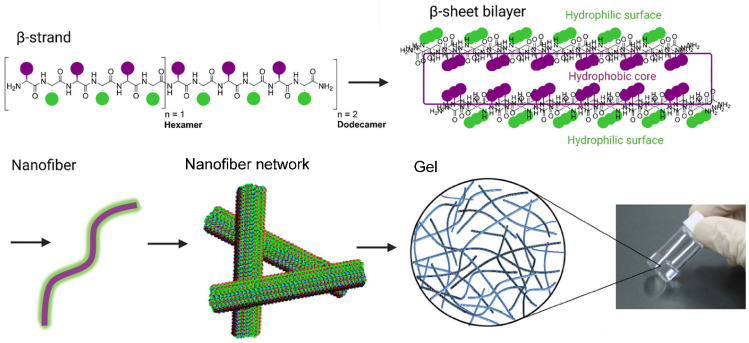
Three-dimensional network formation of peptide hydrogels. Reprinted with permission from Refs. [24,53,184]. Copyright 2020 MDPI, 2012 and 2022 Elsevier.

Amphiphilic peptides, such as KFE8 (Ac-FKFEFKFE-NH_2_), self-assemble in aqueous solutions adopting a β-sheet conformation, as observed with H-FQFQFK-NH_2_ and the doubled hexamer H-FQFQFKFQFQFKNH_2_ (dodecamer) (Figure 27) [24,138,189]. The *π*–*π* stacking of the aromatic residues (phenyl groups) trigger the start of aggregation of the extended β-strands forming a β-sheet bi-layer thus shielding the hydrophobic residues from the aqueous environment [24,132,184,189]. The β-sheet bilayer self-assembles into nanostructures, in this case, nanofibers (1D aggregates), as shown in Scheme 8 [21,138,183]. The β-pleated sheets are recognized as the main contributor to nanofibers and nanotube formation [53]. The hydrophilic residues are exposed to the aqueous environment and participate in electrostatic interactions because of the presence of charged amino acids, which allow for the interaction between fibers and ions [132,138,184,189].

**Figure 28:**
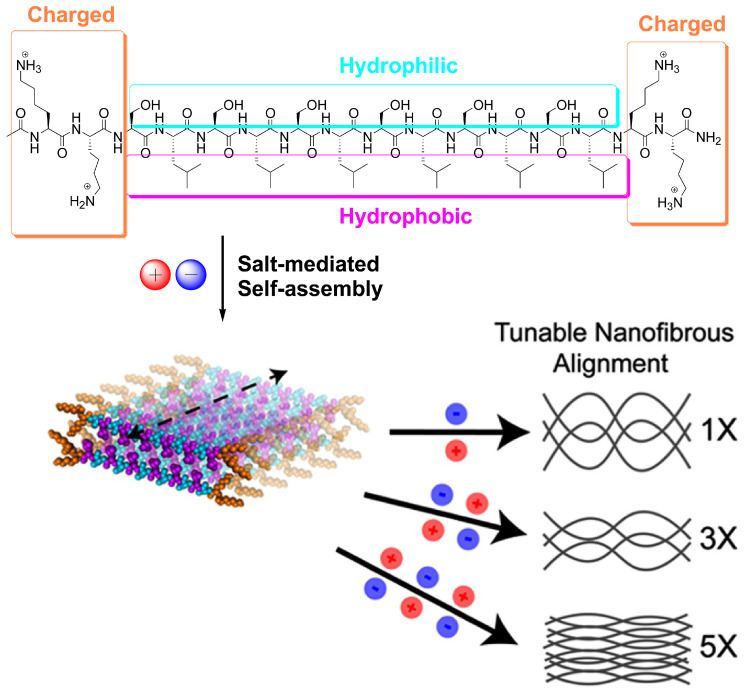
Influence of ionic strength on self-assembly of K2 peptide. Reprinted with permission from Ref. [194]. Copyright 2024 American Chemical Society.

The charges on the hydrophilic residues influence self-assembly, as they can induce an electrostatic repulsion or attraction, depending on the pH of the media. Therefore, when these interactions are balanced, peptides self-assemble into nanofibers and further into hydrogels [138]. This phenomenon was observed in the K2 amphiphilic peptide of K_2_(SL)_6_K_2_ (Figure 28), which undergoes hydrophobic packing of the leucine residue, giving rise to a β-sheet reinforced by backbone-centered hydrogen bonding, whereas charge−charge repulsion at each terminus opposes assembly [194]. The ionic strength of the media acts as a self-assembling trigger to balance the two competing interactions [194]. At low ionic strength, the K2 peptide does not undergo hydrogelation, and the solution remains in a liquid state [194]. At high ionic strength conditions, the K2 peptide undergoes hydrogelation and transitions from a solution to a hydrogel [194].

**Figure 29:**
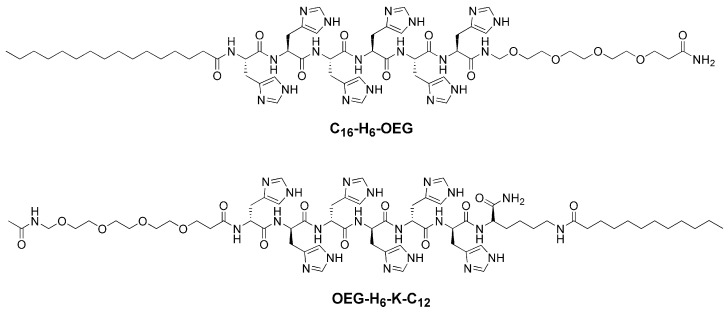
Histidine-based peptide amphiphiles, which disassemble under acidic environments [195].

It was observed by Stupp et al. that changes in pH and concentration can transform the nanobelts into twisted ribbons due to an imbalance of the electrostatic repulsions and hydrogen bond interactions [139]. In addition, changes in pH can result in the protonation of histidine residues in the peptide sequences **C_16_-H_6_-OEG** and **OEG-H_6_-K-C_12_**, resulting in the disassembly of nanofibers under acidic environments (Figure 29) [139,195]. The influence of pH on the peptide folding and self-assembly was also observed by Xing et al., in which, under alkaline conditions, the β-sheets within the self-assembled Fmoc-FF nanofibers transformed into an α-helix [132].

The presence of ions can also induce a salting-out effect, leading to hydrophobic association, which kick-starts the formation of peptide nanofibers and triggers the solution to gel [135]. The presence of ions may also trigger or enhance hydrogelation because the individual peptide nanofibers self-assemble through bundling and establishing physical cross-links amongst each other by hydrogen bonds and electrostatic interactions among the nanofibers, forming a 3D network of peptide hydrogels stabilized by non-covalent forces [21,177,183,184,196,197]. Ionic strength increases the mechanical robustness of hydrogels by increasing the cross-links of polymer chain entanglements [198].

**Figure 30:**
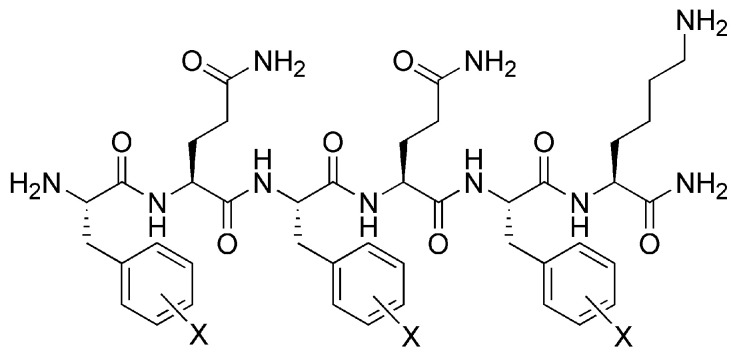
Halogenated phenyl groups (X = halogen) in a peptide sequence to influence the strength of peptide hydrogels [200].

The π−π interactions between the phenyl groups within the hydrophobic core of KFE8 (Ac-FKFEFKFE-NH_2_) and H-FQFQFK-NH_2_ have been found to strengthen the β-sheet-rich fibers (Figure 27), thus stabilizing and enhancing the mechanical strength of peptide hydrogels [132,140,184,185,189,199]. Non-aromatic peptides formed weaker hydrogels than those containing aromatic moieties [135]. Halogenated aromatic groups, such as halogenated phenylalanines, within hydrogel-forming peptides (Figure 30) were found to further increase the strength of peptide hydrogels, in comparison with those shown in Figure 27 by enhancing the affinity between the aromatic groups [200]. Halogenated phenylalanines lead to stronger stacking interactions with heavier halides (F < Cl < Br < I), thus increasing the mechanical strength of the peptide hydrogel in comparison with the aromatics substituted with lighter halides (F) [200].

**Figure 31:**
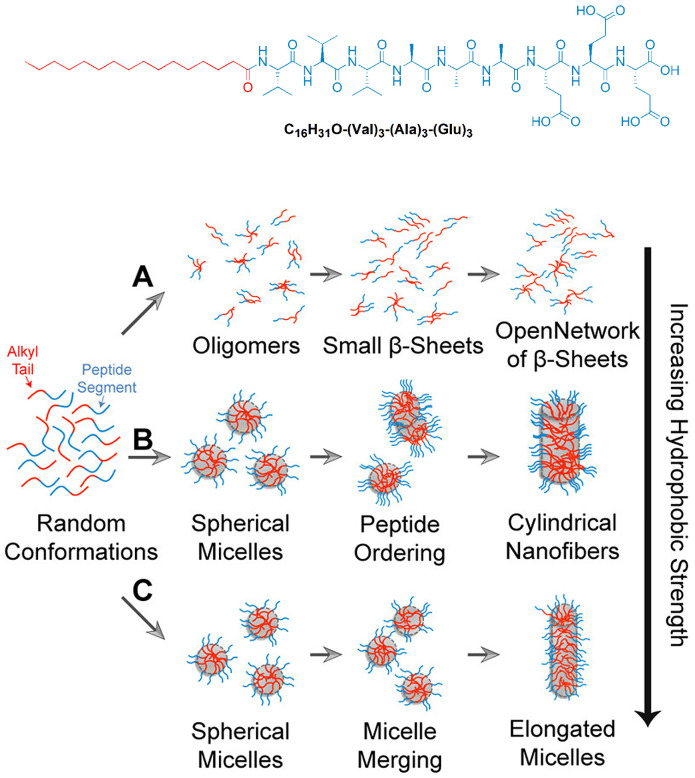
Self-assembly kinetic mechanisms proposed by MD simulation of amphiphilic peptide C_16_H_31_O-(VaI)_3_-(AIa)_3_-(GIu)_3_ in aqueous solution (**A**) at weak hydrophobic interaction strength networks of β-sheets are formed; (**B**) at moderate hydrophobic interaction strength cylindrical nanofibers are formed; (**C**) at strong hydrophobic interaction strength elongated micelles are formed. Reprinted with permission from Ref. [202]. Copyright 2015 Langmuir.

The mechanical properties of hydrogels are important for structural support and functional purposes [201]. However, Nguyen et al. have shown that increasing the hydrophobic strength and reducing the hydrogen bonding ability leads to structural transformations of the aggregates from an open β-sheet network and nanofibers to elongated micelles (Figure 31) [139,202].

### 7.6. Lego Game: Understanding the Influence of Functional Groups on the Gelation of Hydrogels

**Figure 32:**
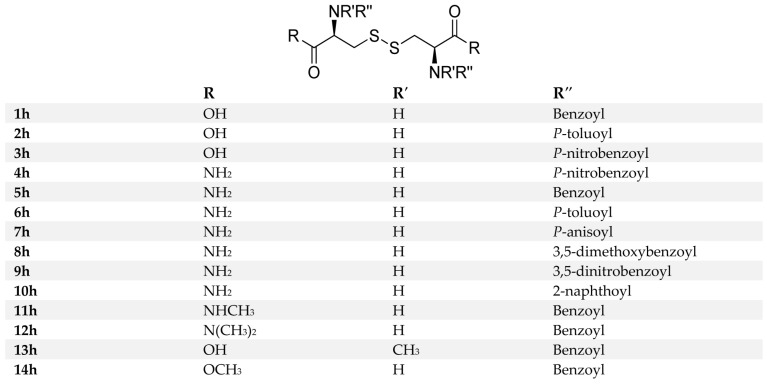
A family of dibenzoyl-L-cystine (**1h**) derivatives demonstrating hydrogelation properties [203].

In 1921, Gortner and Hoffman discovered that dibenzoyl-L-cystine (**1h**) forms a strong aqueous gel. Several decades later, 14 derivatives (Figure 32) were synthesized as potential gelators in an attempt to understand the influence of various functional groups on gelation. This study serves as a good example of a systematic approach to investigate the influence of the substituents on an amino acid-based gelator. The carboxyl protons in **1h** were found to have no significance on gelation efficiency, as **14h** was able to gelate in the presence of an ester. Gelators **4**–**7h**, **10h**, and **11h** are amides of the carboxyl group (**R**) and were found to gelate at a faster rate. It was also observed that amides tend to be less water-soluble than the parent gelator **1h**. Gelator **10h**, with primary amides and naphthoyls, was found to be the best gelator in the series; this could be due to the π–π stackings of the large naphthalene groups stabilizing the gel fibers [203].

Gelator **13h** formed crystals, which showed a linear stacking with each unit connected to the one above and below it by two hydrogen bonds each. The amide-NHs serve as hydrogen-bond donors, whereas carboxyl carbonyls serve as the hydrogen-bond acceptor. The aromatic rings are situated one above another through π–π stacking, which further contributes towards the fiber’s stability. It was assumed that if a similar packing pattern existed in a gel, then a stronger gel could be produced by accentuating the hydrogen-bonding acceptor capabilities of the carbonyl. However, neither the highly electron-rich **8h** nor the electron-poor **9h** were found to be good gelators. Such behavior was not expected, and it could be due to the delicate balance between gelation and crystallization [204]. As Brandon stated in 1920, gelation is an incomplete crystallization, and LMW gels are a solid-like network having a degree of crystalline order with a highly mobile liquid-like phase [203,205].

### 7.7. Self-Assembly of Peptide Hydrogels at the Molecular Level

The mechanism that leads to distinct network morphologies during the self-assembly of peptide-based hydrogels is still being investigated in order to better understand how the intricate balance of different weak intermolecular interactions influences the process of gel formation [189]. Computational methods have been used to reveal the relationship between molecular chemical structure and self-assembly processes as well as assembled morphologies, while high-resolution atomic force microscopy has been used in visualizing and unraveling the molecular organization of self-assembling peptide-based hydrogels on a molecular level [189,206].

**Figure 33:**
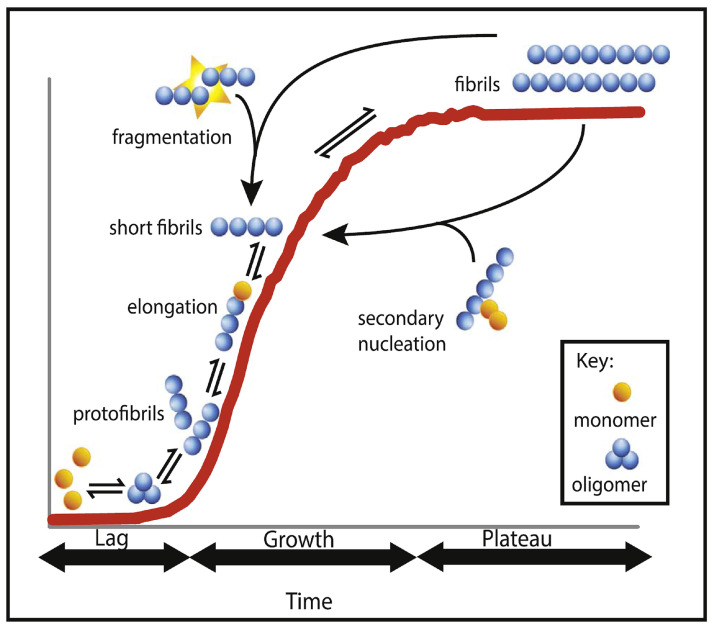
Different phases during fibril formation as progressed over time from the monomer phase to fully assembled phase. Reprinted with permission from Ref. [211]. Copyright 2017 Elsevier.

Thermodynamic and kinetic studies are important in understanding the peptide gelation process [198]. The kinetics of self-assembly for fibril formation proceeds from the lag phase to the growth phase and, finally, the plateau regime (Figure 33) and is dependent on the ability to overcome energy barriers to transition between different phases [198,207]. At time zero during the self-assembly process, peptide monomers and small amounts of oligomers are only present, and they undergo a structural rearrangement process to form a molecular aggregate (nucleus) leading to ordered protofilament (seed), which co-exist with monomers; this process is termed primary nucleation [93,189,208,209,210].

In classical nucleation theory, the small aggregates of monomers have high interfacial energy towards water; the individual monomers that assemble to form an aggregate have a high probability of dissociating back to their component monomers [208,212]. However, some aggregates, such as critical nuclei, can persist and eventually mature to act as a seed for fibril growth [211]. Critical nuclei are the smallest aggregates with the highest Gibbs free energy and grow faster by monomer addition than they dissociate back to smaller aggregates and monomers [208,213].

The self-assembled aggregates emerge through the spontaneous association of monomers [208]. This process of seed formation (fibril nucleation) is typically slow, characteristic of a lag phase with a high free energy barrier [208,210,214,215]. The concentration of free monomers is approximately constant because they are not depleted by growing aggregates during primary nucleation [208,216]. Primary prefibrillar aggregate starts fibril formation independent of previously formed fibrils [189]. This process involves only monomers. It may be a homogenous reaction that is monomer-dependent for new fibril formation or may be heterogeneous, in which the formation of a nucleus is catalyzed by a surface, such as a vessel wall, or any surface present in the system: a phospholipid membrane, nanoparticles or the air-water interface [93,213,217,218].

Once a protofilament or protofibril has reached a critical size, it can serve as a template for the addition of more peptide monomers towards the end of preformed fibrils, supporting fibril growth via elongation. This process is referred to as secondary nucleation. It results in the formation of mature fibrils followed by a fast growth phase when the fibril mass concentration increases rapidly [93,209,210,214]. Secondary nucleation exists in the presence of a seed aggregate of monomers. Therefore, secondary nucleation is not the same as heterogeneous primary nucleation; however, they both speed up the aggregation process [213]. Formation of new fibrils can also be catalyzed by existing fibrils, whereby monomers add to the end of existing fibrils or through fibril fragmentation that forms multiple small fibrils, which have growth sites that serve as nucleation points, thus exponentially increasing fibril mass during fibril elongation [189,209,218]. Fibril fragmentation may be due to a number of factors, such as thermal fluctuations or mechanical stress [211]. During the growth phase, the free monomers become depleted as the aggregate mass approaches saturation [216]. The rate of secondary nucleation is dependent on the concentration of both monomers and fibrils in solution, while fragmentation is a monomer-independent process, which depends only on the concentration of fibrils [93].

The morphology of the KFE8 (Ac-FKFEFKFE-NH2) peptide studied on the solid-liquid interface using AFM on a mica surface revealed that fibril formation was catalyzed by existing fibrils [189,219]. Furthermore, fibril formation for the peptide hydrogelator SgI_37-49_ was also observed to be governed by the secondary nucleation of monomers on the surface of existing fibrils [93]. This observation was supported by a simulation study, which found that the number of β-sheets per fibril increases over time, indicating that already formed β-sheets are being added to the fibril’s side [214]. The kinetics of fibril formation (secondary nucleation) depends on a range of physicochemical parameters, such as peptide concentration, solvent, ionic strength, pH, temperature, and the presence of a surface [93,210]. The increase in fibril mass concentration is typically driven by the growth of existing aggregates, which occurs through the elongation of protofibrils and fibrils by the addition of monomers to their termini [93]. The rate of secondary nucleation quickly becomes much greater than that of primary nucleation [93]. As the total mass of aggregated peptide increases throughout the lag phase, the rate of the fibril-catalyzed secondary nucleation process increases exponentially [93]. The growth phase consists of the rapid consumption of the available monomer and a concurrent increase in total aggregate mass [93]. Monomeric peptides are quickly consumed during the growth phase, causing the rate of primary nucleation to fall and secondary nucleation to dominate [214]. The rate of secondary nucleation, which is heavily dependent on monomer concentration, reaches its maximum shortly after the start of the growth phase [93].

Primary nucleation involves monomers only and may occur in bulk or on a foreign surface [213]. The influence of surfaces on self-assembly is another factor that must be considered in the kinetic studies of hydrogel fibril formation, as evidenced by the heterogeneous nature of SgI_37-49_ primary nucleation [93]. Surfaces have been shown to be capable of accelerating and retarding the self-assembly of peptides, as was observed by the AFM on the self-assembly of KFE8 (Ac-FKFEFKFE-NH2) on a mica surface versus in bulk solution [93,189]. Fibril formation of KFE8 (Ac-FKFEFKFE-NH2) on the mica surface started rapidly after the injection of the peptide solution due to the presence of a nucleation seed, adsorbed to the mica before injection of new material, showed to initiate fibril formation, and a network was formed rapidly without intermediate morphologies [189]. While in bulk solution, a globular peptide aggregate of different sizes was the first observed structure possibly comprised of peptide aggregates in an unorganized manner, leading to the formation of higher ordered aggregates and intermediate morphologies at a slower rate as observed when helical ribbons transitioned into nanotubes [189]. This was also confirmed by MD simulation calculations of free energy by Sayar and Hess et al. upon the absorption of amphiphilic peptides from bulk to the air–water interface. It was found that there is competition between the dehydration of the hydrophobic side (driving force) and the loss of orientational degrees of freedom of amphipathic peptides at the air–water interface (opposing force) [139]. The presence of an air–water interface could assist in distinguishing the hydrophobic and hydrophilic domains, resulting in stable peptide structures, thus facilitate peptide self-assembly [93,139].

Still, there are a number of factors that present challenges for the development of mechanistic descriptions of hydrogel self-assembly, such as the molecular mechanism of the two nucleation processes [93,213]. Understanding the mechanism of secondary nucleation at a molecular level is still elusive [215]. The development of kinetic models of filamentous self-assembly that account for changes in viscosity and diffusion rate during the course of aggregation would greatly facilitate mechanistic investigations of hydrogel fibril formation [93]. The molecular events underpinning the early stages of transition from peptides into fibrillar structures and subsequent growth have remained challenging to elucidate [207]. Focus has also been on fibril formation without considering the effects of gelation on self-assembly, and It has been shown that the two processes can function concurrently [93].

An understanding of the distinct morphology of peptide hydrogels has broad application as they spontaneously self-assemble into amyloid-like fibrils, which are found in neurodegenerative diseases, such as Alzheimer’s disease and Parkinson’s disease [220,221,222]. Therefore, peptide hydrogels are often used as in vitro models for amyloids [223]. This is possible because, at the atomistic length scale, amyloid fibrils are similar to peptide-based hydrogels due to the β-sheet-rich fibrillar structure [185,189,212,222]. The mechanisms through which amyloid-β causes neurotoxic effects are still under heated debate. It is considered that self-assembling peptide hydrogels can be used to elucidate the mechanism through which amyloid aggregates disrupt normal neuronal phenotype and function [212,221,222]. Therefore, understanding the self-assembly of peptide hydrogels can also help with the elucidation of the role of amyloids in neurodegenerative diseases.

## 8. Non-Canonical Amino Acids and Their Applications in Peptide Hydrogels Results

The best gelators are compounds that, although poorly water-soluble, are not so insoluble that they precipitate or crystallize rapidly [203]. Fine-tuning of molecular structures may be needed to achieve the best gelating properties. Non-canonical amino acids (NcAAs) have been exploited to increase the number of available peptide building blocks, and this way to improve and fine-tune hydrogel formation by enhancing secondary structures, which should ultimately result in higher gel stability [162,183,224]. The incorporation of non-canonical amino acids into a peptide sequence with their broad diversity of side chains leads to peptides that self-assemble into various nanostructures with a great diversity of architecture, chemical function, and a high degree of tunability [102,157,170].

### 8.1. Natural and Synthetic Non-Canonical Amino Acids

**Figure 34:**
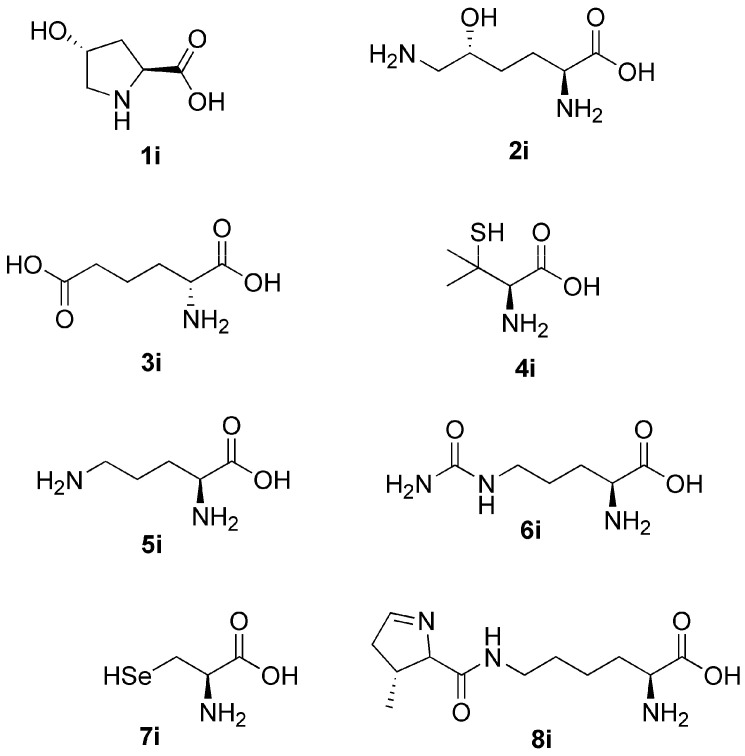
Representative examples of non-canonical amino acids [162].

There are over 120 ncAAs found in natural proteins; however, they are not directly encoded by the genetic code and, hence, are non-proteogenic [161,225] (cannot be directly included in protein molecules during the translation process). NcAAs are often secondary metabolites of canonical amino acids (Figure 34). NcAAs may also be synthesized in the laboratory using the asymmetric Strecker reaction or through direct chemical modification of canonical amino acids, in which reactive cysteine and lysine are mostly used due to their strong nucleophilicity [160,162,192,226,227]. NcAAs impart distinct chemical and biological properties, such as greater stability in vivo, because the enzyme-based chemical machinery in living organisms is tailored to work with the common canonical amino acids [227]. As such, this motivated extensive research into the development and refinement of efficient methods for their preparation [228].

### 8.2. Synthesis of Non-Canonical Amino Acids

**Scheme 9:**
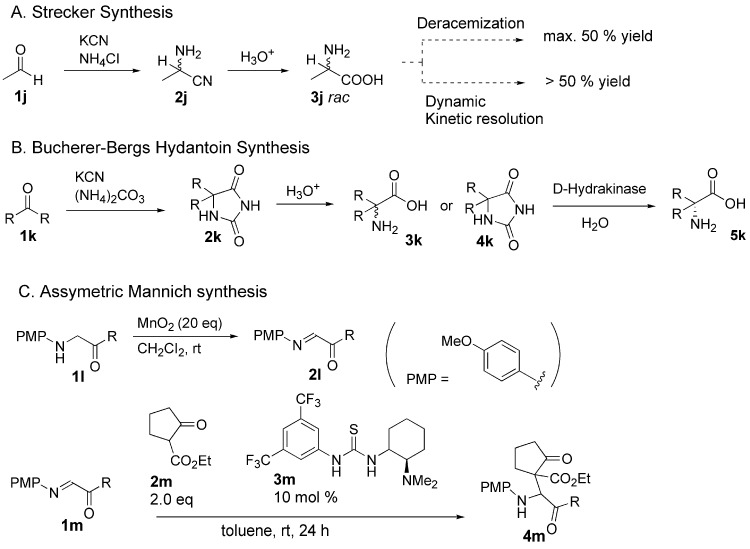
Synthetic methodologies for ncAAs [192,229].

**Scheme 10:**
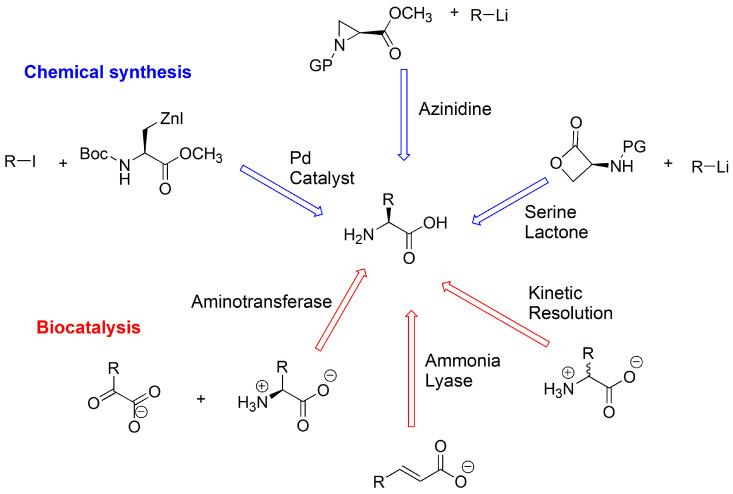
Possible approaches for ncAAs synthesis [227].

Currently, there is a multitude of chemical pathways that prepare ncAAs. The classic Strecker reaction was developed in 1850, and it was the first synthetic approach to (racemic) amino acids [228]. It was carried out by the condensation of aldehydes, ammonia, and hydrocyanic acid-producing amino nitriles, which were converted to amino acids by acidic hydrolysis [228]. The classical Strecker reaction yields racemic products, while Bucherer–Bergs synthesis with enantioselective enzymatic kinetic resolution by hydantoinases yields enantiopure ncAAs [229] (Scheme 9). The use of enzymes has emerged as a versatile approach for the synthesis of ncAAs, and several other approaches have been presented [227] (Scheme 10).

Among the myriad of methods for the synthesis of ncAAs, the asymmetric Mannich-type reaction of α-imino carboxylic acid derivatives is a common strategy because a wide variety of ncAAs can be accessed by changing only the nucleophile (Scheme 9), in which α-imino carboxylic acids is generated by oxidizing N-PMP glycine ethyl ester **1l** with MnO_2_, giving rise to the desired imino ester **2l**, which is coupled with an asymmetric Mannich-type reaction by adding the nucleophile (1,3- dicarbonyl compound **2m**) and bifunctional amino thiourea catalyst in a one-pot reaction [192].

**Scheme 11:**
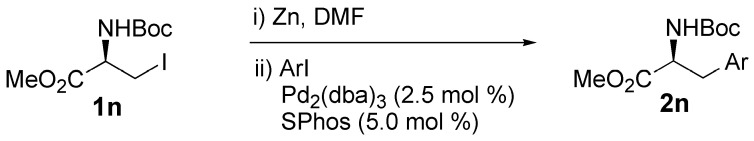
Negishi cross-coupling reaction with a protected iodoserine derivative [230].

In particular, Negishi cross-coupling reactions of halo-serine derivatives allow access to a wide variety of chiral ncAAs using affordable serine as the starting material [230]. Negishi cross-coupling reactions allow two, often readily available, halogen-containing building blocks to be coupled together through a halo-zinc intermediate in the presence of a palladium catalyst (Scheme 11) [230].

Last but not least, ncAAs can be prepared by the attachment of substituents to the canonical amino acids with reactive functional groups, such as lysine (-NH_2_), serine (-OH), aspartic and glutamic acids (-COOH) using standard methods of amide–ester synthesis and appropriate functional group protection–deprotection strategy.

### 8.3. Application of Non-Canonical Amino Acids in Hydrogelators

Interest in ncAAs has emerged due to their applicability within the attractive scaffold of natural peptides, which is widely applied in the field of synthetic biology, pharma, bio-imaging, biosensors, medicinal applications, and organocatalysts design [227,229]. NcAAs are widely used to increase the number of chemical functional groups in canonical amino acids, which constrains the bioactivity of peptides [231]. The significance of ncAAs was demonstrated above (Figure 30) when the incorporation of halogenated phenylalanine into the hexapeptide hydrogelator improved the mechanical strength of the peptide hydrogel.

**Figure 35:**
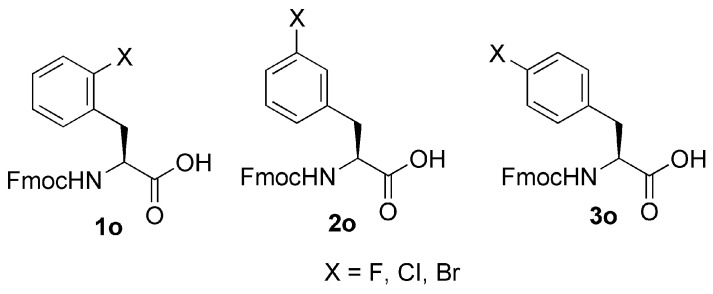
Halogenated Fmoc-Phe hydrogelators [223].

Fmoc-Phe does not readily self-assemble in water; however, monohalogenated (F, Cl, Br) derivatives of Fmoc-Phe (Figure 35) were found to enhance the efficiency of self-assembly of these amino acid derivatives (relative to Fmoc-Phe) to promote hydrogelation in aqueous solvents [223]. The position of halogen substituents (*ortho*, *meta*, *para*) and the nature of halogen itself (F, Cl, Br) influenced the rate of self-assembly and the mechanical strength of the hydrogel [223].

**Figure 36:**
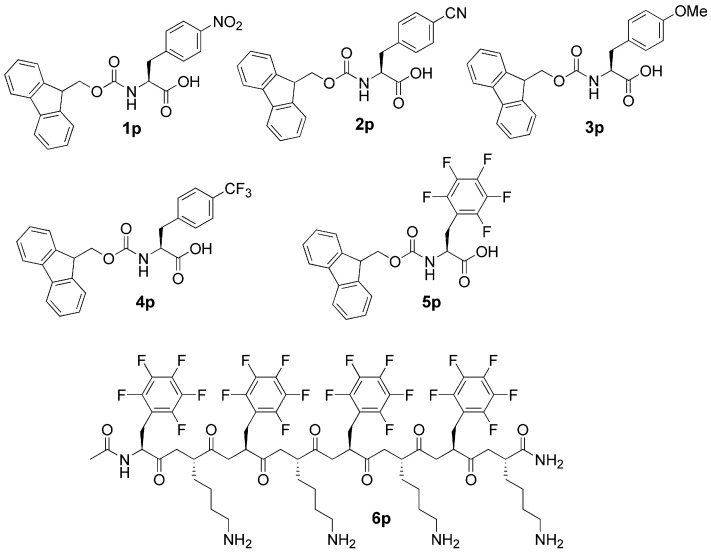
Fmoc-Phe derivatives [183].

The group of Bradley L. Nilsson studied the impact of substitution in the para position of Fmoc-Phe by a nitro (NO_2_), a cyano (CN), a methoxy (OMe), and a trifluoromethyl (CF_3_) on hydrogelation (Figure 36) [183,223]. All these para substitution modifications improved the mechanical strength of the resulting gels, especially for Fmoc-(*p*NO_2_-Phe) (**1p**). Fmoc-pentafluorophenylalanine (**5p**) was found to also undergo hydrogelation in aqueous solutions at accelerated rates relative to that of Fmoc-Phe. In this way, the use of a pentafluorinated phenylalanine (Fmoc-F_5_-Phe) (**5p**) was considered in an oligopeptide hydrogelator (**6p**) [183].

NcAAs were also incorporated in antimicrobial peptides (AMPs) and were found to increase the bioavailability, organism selectivity, metabolic stability and control of charge distribution over natural AMPs [170]. The incorporation of non-canonical fluorinated amino acids into peptides and proteins has been shown to increase their enzymatic stability [230]. The enhanced peptide resistance against chemical and enzymatic degradation is due to the pronounced helix, which is responsible for membrane destabilization and is promoted by the presence of ncAAs [228].

**Figure 37:**
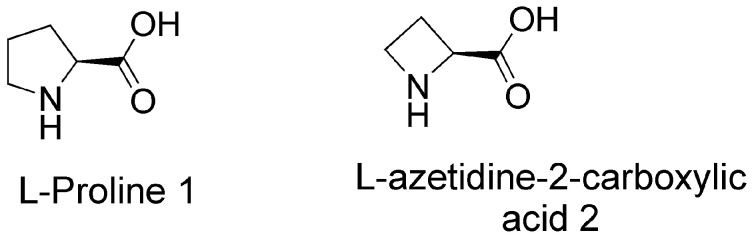
L-azetidine-2-carboxilic acid found in sugar beets [232].

NcAAs were found to be capable of acting as enzyme inhibitors; for example, α-methyl-4-carboxyphenylglycine (M4CPG) was the first compound to express antagonist action at group II and group III metabotropic glutamate receptors [228]. Therefore, ncAAs should be leveraged to access the seemingly endless source of amino acid chemical diversity [158]. However, the use of ncAAs can sometimes result in proteins with improper functions. For example, L-azetidine-2-carboxilic acid (Figure 37) found in sugar beets is a toxic homolog of proline that, if consumed during pregnancy either in pure form or incorporated in a peptide, is believed to cause sclerosis in newborns [162,232].

## 9. Conclusions

Smart drug delivery using nanocarriers has offered an approach to improve the effectiveness of therapeutic drugs. Among the other prolonged drug delivery systems, physical peptide hydrogels represent the class of rapidly developing nanocarriers with high application potential due to their biocompatibility, ease of preparation, and drug release profiles. Most hydrogels have a self-healing character, which is desirable as it enables them to be deformable. However, this character is often associated with low mechanical strength. Therefore, a number of new approaches are currently being investigated to improve the mechanical strength and drug loadings, such as the incorporation of ncAAs, which in most cases is aimed at improving the hydrophobic interactions between peptide chains in hydrogels. Mechanical strength should not be increased at the expense of the self-healing character of hydrogels, as such will result in brittle hydrogels crystallizing or precipitating out of solution.

The morphology of the peptide hydrogel network determines the mechanical properties; however, the mechanism behind the hierarchical self-assembly and cross-linking leading to the formation of the hydrogel network is still not yet well understood in fine detail and is currently under investigation by various research groups. Our understanding of how the morphology of peptide hydrogels emerges during the self-assembly process will lead to an ability to design and create peptide hydrogels with specific qualities of interest. In particular, this knowledge should result in the development of a computational model that will allow the predesign of desired hydrogels, predict the hydrogels’ characteristics, and obtain hydrogels that fit the required criteria.

Much has been learned, developed, and improved about hydrogels since their discovery. Their practical application in several research areas, such as drug delivery and tissue engineering, is constantly increasing, and active research is aimed to further improve and functionalize hydrogels as well as widen their practical application.
